# Bioluminescence in Clinical and Point-of-Care Testing

**DOI:** 10.3390/bios15070422

**Published:** 2025-07-02

**Authors:** Sherwin Reyes, Raymarcos Rodriguez, Emre Dikici, Sylvia Daunert, Sapna Deo

**Affiliations:** 1 Department of Biochemistry and Molecular Biology, University of Miami Miller School of Medicine, Miami, FL 33136, USA; sxr1145@miami.edu (S.R.); edikici@med.miami.edu (E.D.); sdaunert@med.miami.edu (S.D.); 2 The Dr. John T. McDonald Foundation Bionanotechnology Institute, University of Miami, Miami, FL 33136, USA; 3 Nicanor Reyes Medical Foundation, Institute of Medicine, Far Eastern University, NCR, Quezon City 1119, Philippines; 4 Clinical and Translational Science Institute, University of Miami, Miami, FL 33136, USA

**Keywords:** bioluminescence, point-of-care, clinical diagnostics, metal organic frameworks, encapsulation

## Abstract

Point-of-care testing (POCT) offers a transformative approach to diagnostics by enabling rapid and accurate results at or near the site of patient care. This is especially valuable in critical care, emergency settings, and resource-limited areas. However, one major limitation of POCT remains its analytical sensitivity, particularly in detecting low concentrations of analytes. To address this, various innovations are being explored, including advanced sensors, signal amplification, and sensitive labels. Among these, bioluminescent proteins have gained attention for their high sensitivity, fast readout, minimal background interference, and simplified instrumentation. Bioluminescence—light emission from biochemical reactions—presents an ideal platform for enhancing POCT sensitivity. In parallel, metal–organic frameworks (MOFs), especially structures like ZIF-8, are emerging as valuable materials in biosensing. Their high porosity, tunable surface properties, and ability to host biomolecules make them excellent candidates for improving analyte capture and signal transduction. When integrated with bioluminescent systems, MOFs can stabilize proteins, concentrate targets, and enhance overall assay performance. This review highlights the role of bioluminescent proteins in medical diagnostics and their application in POCT platforms. We also discuss the potential synergy between MOFs and bioluminescence to overcome current sensitivity limitations. Finally, we examine existing challenges and strategies to optimize these technologies for robust, field-deployable diagnostic tools. By leveraging both the natural sensitivity of bioluminescence and the structural advantages of MOFs, next-generation POCT systems can achieve superior performance, driving forward diagnostic accessibility and patient care outcomes.

## 1. Introduction

Point-of-care testing (POCT) refers to medical diagnostic tests performed at or near the site of the patient’s care or the doctor’s office, delivering immediate results that significantly increase clinical decision-making and patient outcomes [1,2,3,4]. One of the major strengths of a point-of-care diagnostic device is its ability to deliver rapid information that can lead to timely diagnosis, disease management, and quicker therapeutic interventions. These point-of-care tests have revolutionized patient care in critical care settings, emergency rooms, and remote or underserved areas where access to centralized laboratory facilities is limited [5,6,7,8].

However, one of the major drawbacks of a point-of-care device is its analytical sensitivity, i.e., at how low a level can an analyte be detected. Several research groups are actively trying to improve the sensitivities of these devices by employing novel strategies such as new sensor technologies, signal amplification strategies, improving reagent qualities, using advanced materials, or using sensitive labels [9,10,11].

One of the more sensitive labels that can help increase the sensitivity of the point-of-care test is the use of bioluminescent proteins. Bioluminescence, the emission of light by chemical reaction, provides the end user with several advantages, such as high sensitivity, rapid readouts, simplified measuring modalities, and minimal background interference [12,13,14]. These capabilities have enabled the easy visualization of protein–protein interactions through energy transfer detection [15], molecular and cellular processes using charged-coupled device (CCD) cameras [16], use of bioluminescent reporter in bioanalysis and sensing, and measuring drug concentrations without extensive laboratory equipment [17]. Several reviews have been published recently that discuss bioluminescence and its versatile application from biochemical to environmental applications [18,19,20,21,22]. However, none of them have focused specifically on POC diagnostics. This manuscript will discuss the use of bioluminescence from a point-of-care perspective and discuss its advantages, applications, disadvantages, and other considerations.

## 2. Bioluminescence and Its Natural Occurrence

**Figure 1:**
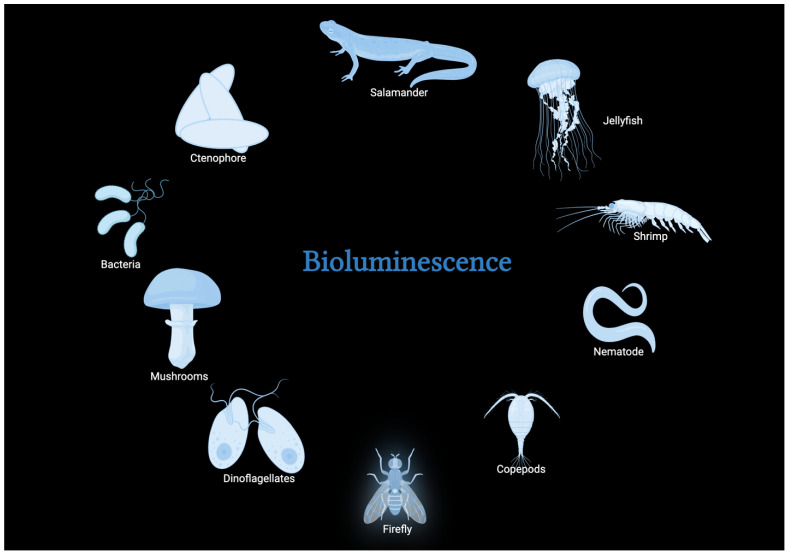
Bioluminescence in nature.

The emission of light produced by living organisms, known as bioluminescence, has attracted the attention of curious viewers and scientists. Bioluminescence can occur in many forms of life, from the smallest unicellular organisms up to the larger forms of life (Figure 1). Bioluminescent animals, found in various habitats such as the depths of oceans and the concealed nooks of forests, produce radiant displays of light that serve as a tribute to the extraordinary array of life forms on our planet [23,24]. The captivating natural occurrence under discussion is not solely a visual spectacle but a multifaceted interaction of molecular mechanisms, wherein bioluminescent proteins have a prominent role in coordinating light emission [25]. In addition to its captivating visual appeal, bioluminescence has become an asset in diagnostic medicine [26], providing valuable information regarding cellular mechanisms, the advance of diseases, and the effectiveness of treatments [27]. This section of this review will focus on the complexities of bioluminescence by discussing bioluminescent proteins, progress made in their use in the medical field, specifically point-of-care testing, and the challenges to be solved.

Bioluminescence can be traced back to ancient times, spanning millions of years, and is observed as a widespread adaptation among various taxa [28,29,30]. There are numerous functions for which this naturally occurring phenomenon is advantageous to the survival strategies of various organisms. Within the profound recesses of the Earth’s seas, the phenomenon of bioluminescence assumes a paramount role as a crucial means of communication for many marine organisms [31]. This remarkable ability allows these species to convey signals to potential mates, dissuade potential predators, or entice prospective prey. On the other hand, some organisms, such as fireflies, utilize their bioluminescent displays as complex courting rituals in terrestrial habitats, whereas some fungi [32] emit luminescence in the obscurity of forests, potentially facilitating the spread of spores [28].

### 2.1. Bioluminescent Proteins

Bioluminescent proteins, known for their capability to produce light through a chemical reaction, are now fundamental in scientific research and diverse applications. There are several systems that can produce bioluminescence, the most prominent ones being the D-luciferin-dependent system and the coelenterazine-dependent system [33]. Within the D-luciferin-dependent system, the complex mechanism for producing light includes the oxidation of luciferin, a light-emitting molecule, catalyzed by the enzyme luciferase [34]. Notable enzymes that take part are firefly luciferase, a key tool in molecular biology research, and click-beetle luciferases, which tolerate an extensive range of pH conditions and produce a variety of colors [33]. The Renilla luciferase, a well-known enzyme in the coelenterazine-dependent system, catalyzes the oxidation of coelenterazine to produce a blue-green bioluminescence that is derived from the sea pansy, *Renilla reniformis* [33]. Additionally, aequorin is a photoprotein in jellyfish that serves as a calcium indicator in cellular studies. This luminescence is prevalent and can be seen in fireflies, bacteria, fungi, jellyfish, insects, and different marine species (Table 1).

The bioluminescence reaction involves the binding of luciferin to luciferase, facilitated in the presence of oxygen and co-factors. This binding initiates the oxidation of luciferin, leading to the release of light photons. The beauty of bioluminescence lies in its efficiency, as the chemical process produces light without generating heat. This energy-saving method has been developed separately in different species/groups of organisms, highlighting its adaptive advantages [27].

**Figure 2:**
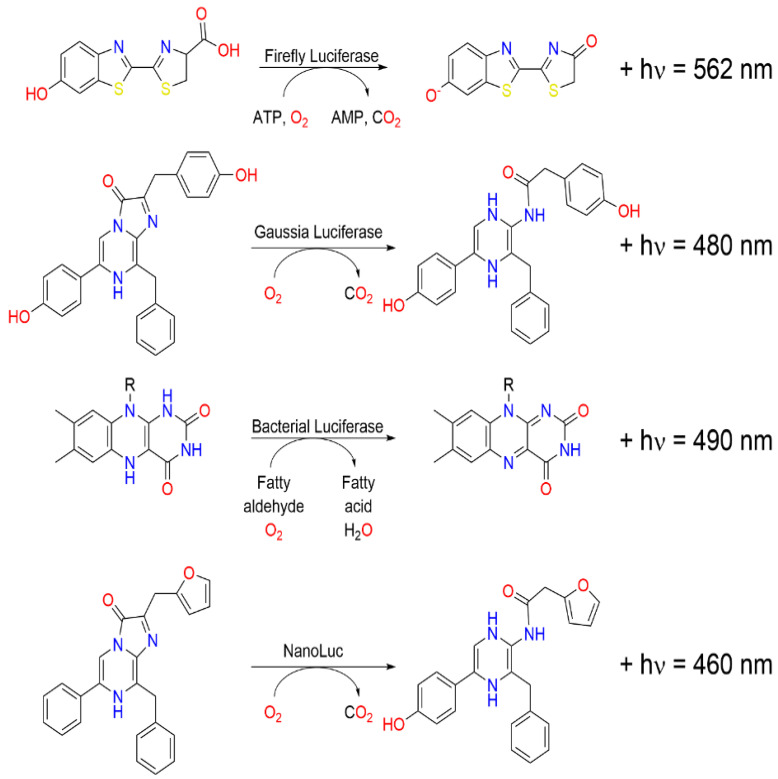
Some representative bioluminescent reactions. The reactions are catalyzed by the luciferin, coelenterazine, and flavin oxidizing classes of luciferase reporter transgenes. The chemical structures of the luciferase substrates and their oxidized products are indicated, together with the wavelength of the emitted light.

The mechanisms of oxidation of D-luciferin and coelenterazine, substrates for *Photinus pyralis* firefly luciferase and Renilla luciferase, respectively (Figure 2), follow very complex metabolic routes culminating in light emission [35]. The pathway of D-luciferin is initiated by the binding of the molecule to firefly luciferase in the presence of ATP and Mg^2+^. Consequently, the newly formed luciferase-D-luciferin-AMP complex reacts with molecular oxygen (O_2_) to produce oxyluciferin in its excited state. While returning to the ground state, this oxyluciferin releases a yellow-green (562 nm) light while forming carbon dioxide (CO_2_) and AMP as by-products. It is this elongated wavelength emission, alongside its aqueous solubility, that allows D-luciferin to undergo in vivo activity with ease. Furthermore, D-luciferin can be synthesized in a direct and cost-effective manner. Studies have demonstrated the substrate can be produced from inexpensive anilines and Appel’s salt [36], as well as through a six-step process in one-pot synthesis using p-benozquinone (p-BQ), L-cysteine methyl ester, and D-cysteine [37], among other methods. Bioluminescent proteins, particularly the one from fireflies, have a high quantum yield, 88% with a range of ±25% or higher [38], for their enzymatic reactions and are therefore reasonably efficient at converting the energy released from a chemical reaction directly into light energy with little loss of energy as heat [39]. Such efficiency is realized through highly optimized enzyme reactions where a luciferase enzyme catalyzes an oxidation reaction with the molecule luciferin to result in photon emission. Optimizations were performed on the conditions of the reaction, such as pH and temperature, and the availability of cofactors such as oxygen. Second, the luciferase protein structure has been engineered to minimize non-radiative decay pathways so that most of this chemical energy is released as light [40]. It seems that evolutionary optimization has further increased the efficiency of these proteins by favoring mutations that improve light emission [41].

In the presence of O_2_, coelenterazine binds with high affinity to Renilla luciferase, leading to the formation of a dioxetanone intermediate. The formed intermediate decays with a concomitant formation of an excited state coelenteramide. Then, coelenteramide decays to the ground state with the release of blue-green light at 480 nm; at the same time, one CO_2_ molecule is formed as a byproduct. Photoproteins such as aequorin and obelin bind calcium undergoing a change in conformation followed by conversion of coelenterazine to coelenteramide, a similar reaction to the bioluminescence emission mechanism observed with Renilla luciferase.

**Table 1:** Examples of a few bioluminescent proteins.

Bioluminescent Protein	Protein Data Bank	Crystal Structure	Source	Molecular Weight	Substrate	Co-Factor	Emission Wavelength	Reference
Bacterial Luciferase	1LUC	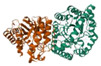	*Vibrio harveyi*	62 kDa	Reduced flavin	FMNH_2_	490 nm	[42]
Firefly Luciferase	1LCI	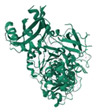	*Photinus pyralis*	60.82 kDa	D-Luciferin	ATP, Mg^2+^	560 nm	[43]
Dinoflagellate Luciferase	1VPR	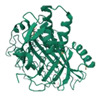	*Lingulodinium polyedra*	42.03 kDa	Luciferin	Oxygen	475 nm	[44]
*Renilla* Luciferase	2PSH	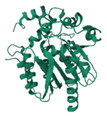	*Renilla reniformis*	36 kDa	Coelenterazine	Oxygen	480 nm	[45]
Gaussia Luciferase	7D20	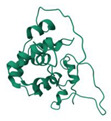	*Gaussia princeps*	20 kDa	Coelenterazine		480 nm	[46]
Oplophorus (NanoLuc)	7VXS	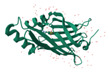	*Oplophorus gracilirostris*	19 kDa	Coelenterazine		455 nm	[47]
Aequorin	1SL8	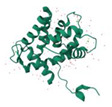	*Aequorea victoria*	21 kDa	Coelenterazine	Ca^2+^	465 nm	[48]
Berovin	4MN0	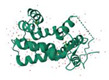	*Beroe abyssicola*	25.47 kDa	Coelenterazine	Ca^2+^	491 nm	[49]

The exceptional efficiency of bioluminescent reactions makes luciferases and their substrates highly desirable tools in a wide range of biochemical and medical research fields [50]. Bioluminescence is greatly valued in a wide array of analyses due to its high sensitivity, rapid/real-time detection, simplicity, versatility, and low-cost features. Bioluminescence can detect very small amounts of target molecules due to the low background noise associated with signal measurement. Bioluminescent assays usually require very minimal sample preparation, making them user-friendly. The value of bioluminescence stems from the fact that it is very sensitive, provides fast results, is simple to use, and is relatively inexpensive to prepare. Therefore, it has been utilized extensively in research. These bioluminescent proteins have transformed the landscape of biological research and have found use in numerous assays and imaging techniques. Specifically, D-luciferin can be chemically modified to function as a pro-substrate. For example, attaching a glucuronide group to the hydroxyl group forms D-luciferin-O-β-D-glucuronide, rendering it inactive until cleaved by β-glucuronidase [51]. This approach allows the monitoring of β-glucuronidase activity, which is often overexpressed in cancer cells or certain bacterial infections. Their natural forms, however, exhibit limitations as to brightness, stability, or specificity that may not meet the demands of advanced applications. Novel sensory configurations, such as split luciferase systems, have been developed with the aim of complementation and chimeric bioluminescent proteins have been engineered for increased signal efficiency [52] This would allow improved sensitivity in molecular imaging and aid in biosensing applications. However, site-directed mutagenesis or site-specific mutations would also help solve some of these challenges [53]. This technique now enables changes in a few amino acids of the protein structure, so its properties can be adapted to specific functions [27].

Site-directed mutagenesis enables such modifications in bioluminescent proteins, including firefly luciferase, Renilla luciferase, and aequorin. It permits tuning the emission wavelength by allowing the color of the emitted light to be modified for multicolor imaging. The proteins may be also engineered to be stable at desired temperature and pH to a certain degree. In addition, substrate specificity and reaction kinetics can be fine-tuned by mutagenesis in order to increase the interaction between protein and substrate, which might have an even higher sensitivity in application to biosensing [54].

Such mutant proteins have enormous applications in biotechnology and medical diagnostics. Optimized bioluminescent proteins are used as ultrasensitive reporters for detecting biological markers. This allows researchers to image living cells in real time to aid in their search for new pharmaceuticals. They can also be altered in such a way that they can respond to pollutants or toxins and then use light as evidence of contamination. For example, in a study completed by Auld, D. et al., 2009, they compared *Photinus pyralis* (lucPpy), a commonly used firefly luciferase, with Ultra-Glo luciferase that directly evolved from *Photuris pennsylvanica* [55]. In the directed evolution, mutagenesis was performed, selecting mutations that led to increased thermal stability, improved signal stability, and reduced sensitivity to inhibitors. After conducting several high-throughput screening (HTS) assays, Ultra-Glo luciferase retained high activity while being less affected by inhibitors, serving as a superior ATP sensor in HTS assays. Thus, the directed evolution optimized the enzyme structure to prevent nonspecific compound interactions, improving assay reliability that could be implemented in POC assays [55]. Overall, site-directed mutagenesis has extended the functional repertoire of bioluminescent proteins and turned them into indispensable tools in a plethora of different research and diagnostic branches [56].

Some examples of bioluminescent proteins with high quantum yields are firefly luciferase, Renilla luciferase, Gaussia luciferase, and aequorin. These high quantum yield proteins have been developed specifically as donors for bioluminescence resonance energy transfer (BRET) reactions. BRET is a sensitive, non-invasive technique for monitoring the interactions between proteins in living cells. In BRET, when the protein bearing the bioluminescent donor and the molecule that bears the fluorescent acceptor are in proximity to each other, energy is transferred. Intrinsic properties with high quantum yield make these bioluminescent proteins of special value for use in applications requiring reliable and efficient emission of light, such as in biotechnology, medical imaging, and point-of-care testing.

Researchers have utilized bioluminescent proteins for various purposes, especially in the field of molecular and cellular biology. These proteins act as valuable reporters in genetic and cellular tests, allowing for the non-invasive and precise tracking of biological processes [16,57]. The ability to observe and analyze in real time has greatly improved our comprehension of complex biological processes. Examples of bioluminescent proteins used include Photinus pyralis luciferase, Renilla reniformis luciferase, Gaussia princeps luciferase, NanoLuc Luciferase, aequorin, and various mutants of these proteins.

Photinus pyralis luciferase is one of the most popular enzymes employed in several reporter gene assays for measuring gene expression, protein–protein interactions, and cellular events, in addition to imaging tumors and biological activities in living animals [58,59,60]. The Renilla luciferase is used in dual luciferase assays for the study of gene expression and activity of promoters in cellular contexts, plus live animal models for imaging gene expression and protein–protein interaction that help in the elucidation of gene function and disease processes. Gaussia luciferase finds application in the highly sensitive and real-time monitoring of biological processes in vivo. Aequorin, being dependent on Ca^2+^ as a cofactor for its bioluminescence activity, has been employed in the design of highly sensitive biosensing applications, especially for Ca^2+^ flux assays. Another example of a commercially available luciferase is NanoLuc luciferase, and it is a small, bright luciferase engineered for enhanced imaging and sensitivity from the marine organism *Oplophorus gracilorostris* [61]. NanoLuc, when paired with its novel imidazopyrazinone substrate (furimazine), produces a glow-type luminescence with a heightened specificity in comparison to either firefly (*Photinus pyralis*) or Renilla luciferases in glow-type assays [61]. Additionally, it proves to be a reliable reporter enzyme with no evidence of post-translational modifications in mammalian cells and can withstand up to 55 °C or in culture medium for >15 h at 37 °C [61]. Over time, derivatives of NanoLuc have come to fruition, such as NanoLuc Binary Technology, NanoBiT, and HiBiT. NanoBiT allow researchers to study protein–protein interactions due to the binary nature of the assay [62]. NanoLuc is split into two inactive fragments: LgBiT (large BiT) and SmBiT (small BiT), which are then fused to two proteins of interest. If the targeted proteins interact, the NanoBiT fragments act as an active luciferase enzyme and with the addition of its substrate, will emit a bright bioluminescent signal. Studies have used NanoBiT to monitor internalization mechanisms, specifically, antibody-mediated internalization, SARS-CoV2 viral infection, and GPCR internalization/recycling, opening doors for drug discovery and drug development [62]. Similarly to NanoBiT, HiBiT fuses to a protein of interest but remains inactive until it binds to an LgBiT, in which it is then reconstituted as an active NanoLuc luciferase enzyme [63]. However, HiBiT provides more insight on protein quantification and has been reported to be useful in antiviral drug screening for hepatitis E virus and influenza A [63,64].

*Photinus pyralis* firefly luciferase was historically used as the bioluminescent reporter of choice, but its ATP dependency, its large molecular weight, and the discovery of other bioluminescent proteins, have limited its use. For example, Gaussia luciferase or nanoluc, which have been characterized as highly stable and sensitive reporters, gained in popularity for use in highly sensitive diagnostic assays for the detection of biomarkers and disease markers. Their use in various fields, ranging from biomedical research to drug discovery for high-throughput screening to assess the effects of medicines in vivo to environmental applications, demonstrate the flexibility and diversity of the bioluminescence technology [26]. In a study conducted by Calabretta et al., they developed an ATP-driven bioluminescence platform using a luciferin–luciferase reaction. They detailed the creation of an ATP-sensing paper, which is fabricated through a novel freeze-drying procedure. This approach ensures that the paper can be both produced and developed efficiently in laboratories equipped with basic technologies such as freeze-dryer, wax, and 3D printers. This sensing papers can be used with various bioluminescence detection tools, including benchtop luminometers or smartphone cameras, eliminating the need for additional, specialized equipment [65].

Despite their great sensitivity, luciferase-based assays have difficulties with complex biological samples because of enzymatic degradation, sample variability, and interference from body fluids (blood, urine, semen, etc.). Techniques like metal–organic framework encapsulation are employed to improve stability and reproducibility. However, regulatory obstacles, biosafety issues, and the high cost or scarcity of substrates are impeding the translation of these technologies to clinical and commercial use. Furthermore, environmental elements like ambient light, temperature fluctuations, and sample contaminants can affect assay accuracy; therefore, standardized procedures and controlled testing settings are necessary to ensure reliability [66].

Ultimately, the fascinating realm of bioluminescent protein transcends the natural occurrence seen in living organisms. Their incorporation into scientific research and diverse applications has opened opportunities for exploration, enabling breakthroughs in understanding biology, advancing medical diagnostics, and fostering innovation in biotechnology. As researchers continue to explore the beauty and wonders of bioluminescence, the potential for novel applications and discoveries remains limitless. In order to expand on the applications of bioluminescence in biological and medical fields, it would be prudent to start with the historical significance of bioluminescence and how it evolved through the years.

### 2.2. Application of Bioluminescence in Biological and Biochemical Research

Bioluminescence has played a crucial role in scientific research and has had several historical significances across different fields, from the identification and extensive studies on luciferase and luciferin which became the key components in bioluminescence to their various application in the field of medicine, industry, and the environment [67]. The discovery of these proteins and their light emission mechanisms has helped scientists understand and apply bioluminescence in research. Bioluminescence has been a valuable tool in studying various organisms, especially in the marine environment [26]. It has provided insights into the behavior, communication, and ecology of bioluminescent species such as jellyfish, fireflies, and certain species of plankton. The isolation and cloning of the genes responsible for bioluminescence has been significant in the field of molecular biology and biotechnology. For example, the discovery of the green fluorescent protein (GFP) from a species of jellyfish (*Aequorea victoria*) in 1962 by Osamu Shimomura, who was studying the bioluminescent phenomena in jellyfish, has revolutionized molecular biology [68]. Both GFP and bioluminescent proteins have been widely used as molecular markers and reporters in genetic and cellular studies. The discovery and development of the green fluorescent protein (GFP) and demonstration of these proteins as biological markers earned Roger Y. Tsien, Osamu Shimomura, and Martin Chalfie the 2008 Nobel Prize in Chemistry. Today, GFP and its derivatives have become an important tool for any molecular and cellular biologist to visualize and track cellular processes in real time. Chalfie and colleagues introduced the use of GFP as a fluorescent tag for gene expression, protein localization within cells, and cellular component dynamics. This opened the door to biological research with a working and versatile method for understanding the living cell from within [69]. Bioluminescence has also been utilized in imaging techniques, allowing the non-invasive monitoring of the biological process in living organisms, tracking disease progression, studying gene expression, and assessing the efficacy of treatment in vivo using bioluminescent markers. In 2005, Sadikot and Blackwell’s study revealed that bioluminescence imaging (BLI) is an effective way of identifying trans-gene expression in disease models, bacterial infections, and tumor advancement. When compared with GFPs, Choy et al., 2003, showed that BLI in vivo was more sensitive and had less background signal noise as mammalian cells do not have endogenous bioluminescence [70]. This is because GFPs suffer from autofluorescence due to their production of light reflection and light absorbance, hindering the detection of low signals [70]. Additionally, since BLI does not require light excitation and solely relies on the chemical reaction with luciferase and luciferin, no photobleaching occurs. If the reaction continues, the light emission will persist, unlike GFPs and fluorescence-based methods that rely on light-induced stress [71]. Overall, BLI is a non-invasive method of studying gene expression in living creatures with potential applications to improve our understanding of many illnesses. Molecular imaging techniques bridge the gap between molecular and cellular biology, and among them, bioluminescence imaging finds a number of applications in biological research such as POC assays [72].

Other than biological sciences, bioluminescence has found its way into field such as environmental sciences or the food industry as well. For example, some bioluminescent organisms, such as the bioluminescent bacteria *Vibrio harveyi* and *Vibrio fisheri* have been used as indicators of environmental health. These organisms are sensitive to specific pollutants such as heavy metals (mercury, cadmium, lead, and copper), organic compounds (pesticides, herbicides, and polycyclic aromatic hydrocarbons (PAHs)), toxic chemicals (phenols, surfactants, etc.), and wastewater contaminants. Monitoring the bioluminescence of these organisms in the presence of different contaminants can provide insights into the presence of these pollutants in water or soil [73]. Similarly, bioluminescence assays can be used in the food industry providing rapid and sensitive detection of pathogens in food samples. The presence of specific organisms can be detected by measuring the bioluminescent signal generated in response to their presence.

Applications of bioluminescence in the food industry are related to quality control, safety monitoring, and research. Bioluminescent biosensors can detect some bacterial contaminants or spoilage organisms in food products [74]. Such detection can ensure the needed safety for food and prolong shelf life. Bioluminescent monitoring systems are routinely used for following up on cleaning and sanitizing processes at food processing sites. They ensure proper sanitization and the maintenance of food safety standards.

Although bioluminescence has uses in many diverse fields as described above, we will focus in the next sections of this review on the use of bioluminescence in the field of medicine specifically as it pertains to diagnostics.

### 2.3. Exploring Bioluminescence in the Field of Medicine

The impact of bioluminescent proteins extends far beyond the confines of laboratory investigations, offering a promising integration with medicine for diagnostic and therapeutic applications [56]. This convergence introduces a revolutionary approach to observing and tracking biological phenomena in real time within living organisms, holding significant implications for understanding disease mechanisms, assessing therapeutic efficacy, and devising innovative therapeutic strategies [27,75].

**Figure 3:**
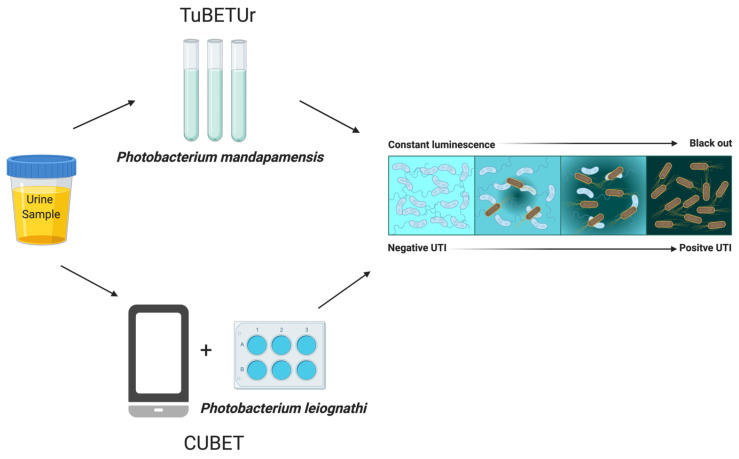
Bioluminescent technologies (TuBETUr: Tube Bioluminescence Extinction Technology Urine and CUBET: Cellphone-based UTI Bioluminescence Extinction Technology) for the detection of urinary tract infection. The figure was reproduced from [78] with permission.

In the realm of diagnostic medicine, bioluminescent proteins and whole cell bioluminescence have emerged as highly valuable resources, enabling non-invasive imaging techniques for detecting and monitoring diseases [76]. Genetically modified cells expressing bioluminescent proteins, when introduced into living organisms, provide a dynamic means for researchers to monitor disease progression and therapy responses over time. Notably, in cancer research, monitoring bioluminescent tumor cells in animal models offers a real-time evaluation of tumor progression, metastasis, and the efficacy of anti-cancer treatments [77], presenting a significant advantage over conventional methodologies that involve sacrificing animals at various intervals for analysis. In a study conducted in our laboratory, two innovative platforms were developed to rapidly diagnose urinary tract infection, namely TuBETUr: Tube Bioluminescence Extinction Technology Urine and CUBET: Cellphone-based UTI Bioluminescence Extinction Technology (Figure 3). These technologies rely entirely on the light extinction of bioluminescent bacteria upon contact with infected urine due to bio-oxygen demand. These tests can be completed within 10 min as compared to standard culture-based methods that require 24 h. The bacteria used in these platforms were marine luminous bacteria: *Photobacterium leiognathi* and *Photobacterium mandapamensis*, making this an inexpensive, sensitive, and user-friendly technology for diagnosing UTI. These bioluminescent bacteria are highly aerobic and require oxygen for their luminescence. Additionally, these specific strains of bioluminescent bacteria can emit light even at lower cell densities, demonstrating high efficiency in bioluminescence-based applications. Notably, these microbes can generate a signal intensity of up to 100 million RLU or more without requiring any external cofactors or substrates. Furthermore, they are easily cultivated in a saline medium, allowing for simple and cost-effective maintenance under laboratory conditions. When in contact with infected urine, these bioluminescent bacteria compete for available oxygen as UTI-positive urine has been previously shown to contain low dissolved oxygen. This competition inhibits the bacteria’s light emission, making the system a reliable indicator of infection. The use of lyophilized whole cells in the system ensures it is inexpensive, highly sensitive, and user-friendly. Additionally, unlike current tests that are based on the indirect monitoring of bacterial products such as leukotriene and nitrite levels, our test can detect and quantitate the load of bacteria, which is important in therapy decisions since bacteria below 100,000 CFU/mL does not warrant antibiotic therapy. This technology is particularly valuable for diagnosing UTIs in low-resource settings, where traditional diagnostic methods may be impractical or costly. These advancements offer a cost-effective and portable solution for rapid UTI diagnosis, utilizing readily available resources and minimal technical expertise, which is ideal for point-of-care settings [78].

This extends to gene expression and cellular dynamics surveillance, where bioluminescent reporters linked to specific genes have become instrumental. This methodology proved invaluable in investigating dynamic phenomena such as circadian cycles [79,80], immunological responses [81], and the initiation of distinct signaling pathways [82,83]. Additionally, bioluminescence imaging finds applications in neuroscience, allowing researchers to delve into brain activity, observe temporal patterns, and understand the functional characteristics of individual neurons or complete neural circuits. This capability significantly contributes to comprehending neurological illnesses and formulating precise therapeutic strategies [84].

Furthermore, assessing treatment efficacy is an important application of bioluminescence in medicine. In this case, researchers leveraged the manipulation of cells to produce bioluminescent proteins linked to specific biological processes, enabling the real-time evaluation of the effects of medications or other therapies [85]. This real-time assessment is of paramount importance in drug development, where the ability to analyze treatment responses promptly can accelerate the testing of potential medicines [77], facilitating the transition of scientific findings from the laboratory to clinical applications [85]. In essence, the versatility and in vivo imaging capabilities of bioluminescent proteins offer a transformative paradigm for diagnostic medicine, paving the way for more effective, non-invasive, and dynamic approaches to understanding and treating various diseases. In a study performed by Sato et al., the authors showed that BLI represents a sensitive and flexible in vivo imaging technique based on the principle of detection of light emission from cells or tissues [86,87]. It has been applied to monitor tumor cells, bacterial and viral infections, gene expression, and therapy response [57,86,87,88]. In vivo bioluminescence imaging enables the real-time, yet non-invasive, monitoring of the progression of disease in a single animal rather than using several animals at different times during the course of the disease. One example is with transgenic mice that are genetically engineered to take up the luciferase gene under the control of the estrogen-responsive element promoter. These animals were used to probe the activation of estrogen receptors. In this case, injection of estrogen-like drugs caused the activation of luciferase activity in vivo in a dose- and time-dependent manner. The model is thus validated as an important tool to study the tissue-specific activity of estrogen receptors and for the screening of estrogenic chemicals [86,89,90]. For example, genetic engineering of luciferase under the control of an androgen-dependent promoter allowed for the identification and study of genes regulated by androgens in mice. In these animals, luciferase expression was increased by testosterone, while anti-androgenic drugs reduced the bioluminescent signal. Bioluminescence was also used in the study of angiogenesis in vivo. This study employed transgenic mice that coupled the luciferase gene to the vascular endothelial growth factor-2 (VEGFR2) promoter to express the VEGFR2 gene in order to track angiogenesis processes during wound healing [86].

### 2.4. Advantages and Limitations of Luciferase-Based POCT Platforms

Luciferase-based point-of-care testing (POCT) platforms are increasingly recognized for their exceptional sensitivity and specificity, particularly when compared to traditional electrochemical and immunochromatographic diagnostic methods. The key advantage lies in the bioluminescence mechanism; these systems can achieve extremely low limits of detection (LOD), often in the picomolar to femtomolar range. This is significantly more sensitive than electrochemical methods, which typically detect in the nanomolar to picomolar range, and immunochromotagraphic assays, whose LODs are generally higher, often falling within the nanomolar to micromolar range. This makes luciferase-based assays especially valuable for detecting low-abundance biomarkers in early disease stages or low-dose exposures.

Another important strength of luciferase-based platforms is their specificity. These systems can accurately tell target analytes apart by using highly selective biorecognition elements like antibodies or aptamers. This reduces the number of false positives. This performance is similar to or greater than that of electrochemical approaches, which rely largely on modifying electrodes to find targets. Immunochromatographic tests, on the other hand, are usually specific, but they can be more likely to cross-react and vary, especially when less tailored antibodies are used. Luciferase assays also do not need outside light to excite them, which reduces background noise and makes the signal clearer, unlike fluorescence-based techniques.

From a practical point of view, luciferase POCT systems are fast, portable, and very accurate. They simply need a few simple tools, such as a small photodetector or a smartphone-based reader, and they can give you results in just a few minutes. This is better than electrochemical platforms, which need more complicated instruments like potentiostats, and lateral flow assays, which are the fastest but usually only give qualitative or semi-quantitative data. Luciferase assays, on the other hand, can be affected by the matrix of the sample or enzyme inhibitors. Immunochromatographic platforms, on the other hand, are made to work with rough biological materials like blood or saliva without any problems.

Luciferase-based POCT platforms have some problems, even though they are good. Their enzymes frequently need to be stored in a cold chain, although this problem is improving because of the continuous work undertaken on enzyme stabilization. They also tend to cost more because it costs money to make enzymes and put them into systems. Immunochromatographic tests are cheap, easy to find, and simple to use, while electrochemical methods are stable and affordable at room temperature. However, luciferase systems are superior at multiplexing, especially when they incorporate more than one luciferase variant or time-resolved signals. In general, their combination of excellent analytical performance, quantitative output, and increasing mobility makes them a strong candidate for next-generation diagnostic applications.

## 3. Point-of-Care in Infectious Disease Diagnosis

Point-of-care (POC) diagnostics have become indispensable in the field of infectious disease diagnosis, offering rapid and on-the-spot testing that significantly improves patient outcomes and public health [91]. By bringing diagnostic capabilities to the patient’s side, POC tests streamline the diagnostic process, allowing for quicker decision-making, timely initiation of treatment, and the effective management of diseases [1]. This approach is particularly critical in remote or resource-limited settings, where traditional laboratory facilities may be scarce, and timely diagnosis is essential for preventing the spread of infections.

Pathogenic microbes, including but not limited to viruses, bacteria, fungi, and parasites, are the primary etiological agents of infectious diseases. Conversely, infectious proteins are also capable of inducing diseases such as Creutzfeldt-Jakob Disease (CJD), kuru, scrapie, and numerous others that are mediated by prions. These infectious diseases have the potential to spread and multiply exponentially within a population in a very short time, posing a significant threat to public safety and impacting the economy. There is a significant threat to humans, as over half of the global population is considered at risk.

Recently, a viral outbreak of a highly contagious respiratory virus Coronavirus disease-19 (COVID-19) was experienced and POC testing played a pivotal role in containing the spread. Various devices and methodologies based on different strategies were employed to contain increasing cases of infections. This includes the following strategies for detecting the virus: POC assays based on nucleic acids, POC detection based on immunoassays, and biosensor-based identification [92]. These strategies were used in mobile healthcare units equipped with POC diagnostic devices. When individuals displayed symptoms, they were quickly tested on-site for the virus using POC assays. Results were obtained within minutes, allowing healthcare teams to promptly identify and isolate infected individuals. This rapid response not only curtailed the further transmission of the virus but also facilitated efficient contact tracing and resource allocation [93,94]. The use of POC diagnostics in this scenario highlights its crucial role in outbreak management, demonstrating its capacity to enhance both individual patient care and broader public health strategies.

### 3.1. Importance of Accurate and Timely Diagnosis

Without diagnostic procedures, medical interventions can be ineffective. It is critical to provide a timely and accurate diagnosis [91]. Efficient treatment and prevention of the transmission of infectious diseases are both significantly facilitated by prompt, critical, and accurate diagnostic testing. Core clinical laboratories offer highly sensitive and specific assays, including mass spectrometry (MS) tests, high-throughput chemical and immunoassays, blood cultures, and polymerase chain reaction (PCR)-based nucleic acid amplification tests (NAAT). These techniques are frequently time-consuming, labor-intensive, expensive, and dependent on specialized machinery and operators. Point-of-care (POC) tests, conversely, offer prompt and localized results, thereby surmounting certain limitations associated with centralized testing methodologies.

### 3.2. Requirements of Point-of-Care Tests

**Figure 4:**
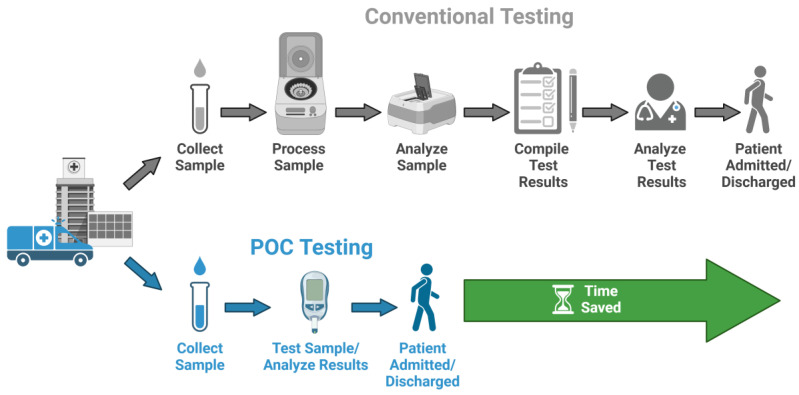
Point-of-care test schematic diagram.

Point-of-care testing (POCT) has emerged as a paradigm shift in the landscape of medical diagnostics, reflecting a transformative approach aimed at providing rapid and accessible diagnostic information at or near the patient’s location of care. The World Health Organization (WHO) defines POCT based on the RE-ASSURED criteria: Real-time connectivity, Ease of specimen collection, Affordable, Sensitive, Specific, User-friendly, Rapid and robust, Equipment-free, and Deliverable [95,96], meaning that POCT is intended to provide rapid, reliable diagnostic solutions without the need for a comprehensive laboratory infrastructure. In this regard, POCT will be apt for low-resource and rural settings. These tests can rapidly diagnose infections, monitor chronic conditions, or identify biomarkers for various diseases, all at the patient’s side, thus improving healthcare outcomes by facilitating immediate treatment decisions. These characteristics highlight the importance and relevance of POCT in modern healthcare settings. POCT fundamentally differs from standard centralized laboratory testing by enabling the quick examination of patient samples near the clinical contact. This immediacy is supported by the necessity for timely clinical decision-making, allowing healthcare providers to promptly start therapies and improve patient outcomes (Figure 4). Point-of-care testing is affordable, making diagnostic capabilities accessible across all demographic groups regardless of economic discrepancies.

The sensitivity and specificity requirements emphasize the importance of point-of-care tests providing precise and dependable results, confirming their significance as essential weapons in the diagnostic arsenal. User-friendliness is an essential aspect that focuses on making tests easy to use and understand. This helps healthcare professionals use the tests effectively and allows individuals with limited medical knowledge to conduct self-tests when necessary.

The quickness and strength of the diagnostic process are crucial in point-of-care situations, where speedy results are essential, and the diagnostic process needs to endure various clinical obstacles. Clinical challenges in point-of-care testing have a significant impact on its effectiveness and reliability. After all, it is difficult to achieve central laboratory performance in the POCT setting due to operator error, environmental conditions, and device limitations, which may lead to misdiagnosis and incorrect treatment decisions. Sensitivity and specificity are very critical to the clinical utility of POCT, since suboptimal levels of either of these parameters could be related to important clinically relevant barriers. Low sensitivity results in false negatives, which lead to missed diagnosis, delayed treatment, and increased complications. On the other hand, low specificity results in false positives, which are associated with anxiety and unwarranted treatment, albeit at a lower cost and strain on healthcare resources. Balanced parameters are necessary to guarantee diagnosis and foster clinical trust. Periodic calibration, maintenance, and device validation are necessary for maintaining quality control and assurance in decentralized settings. This will necessitate the appropriate training of healthcare personnel to prevent test administration and result interpretation errors. Again, accurate result interpretation from POCT requires clinical acumen regarding knowledge of test limitations to prevent misdiagnosis.

Other challenges presented include navigating the maze of regulatory compliance to ensure patient safety and device approval, integrating POCT results into electronic health records to obtain comprehensive care, and effectively managing patient follow-up. The risks of cross-contamination make biosafety and infection control very important, particularly where infectious disease testing is concerned. With a reduced menu in POCT compared to the centralized laboratory, the need for test menu expansion is very important without compromising on accuracy. Assuring access by clinicians to decision support tools, finding a balance between cost and benefits for POCT devices, gaining patient trust and compliance—all these are very important. Overcoming these challenges will require perpetual validation, appropriate training, rigorous quality control, technological advances, and collaboration among health care providers, manufacturers, regulators, and policymakers to develop better, more reliable, and more clinically useful POCT that will benefit patients’ care and outcomes. The lack of equipment for POCT sets it apart by reducing reliance on sophisticated apparatus and promoting flexibility in environments with limited resources or remote locations. The deliverability requirement assures that diagnostic tests can be easily transported and distributed, expanding their availability to geographically dispersed or underserved groups.

In summary, point-of-care testing represents a complex array of clinical urgency, technical sophistication, accessibility, and stringent criteria adherence. Point-of-care testing is an integral part of healthcare to ensure precision therapy, improved patient outcomes, and to expand the availability of diagnostic tools outside traditional healthcare settings.

### 3.3. Technical Strategies to Optimize Light Collection in Bioluminescent POC Devices

Bioluminescent signals in POC settings are frequently faint, necessitating precise light-capture techniques for successful detection. Several optical engineering techniques have been used to improve light gathering. Reflective chambers, which are frequently covered in aluminum or PTFE, are employed to send stray photons toward the detector, which greatly increases the signal strength [97]. Optical lenses and diffusers assist focus and spread out the light that comes out, while bandpass filters are very important for blocking out background noise by isolating certain wavelengths.

Smartphone-based detectors are easy to carry and use, but they have problems with sensor sensitivity, dynamic range, and interference from ambient light. Researchers have solved these problems by using 3D-printed optical housings [98], adding micro-lens arrays, and making their own software to manage and post-process signals.

Integrating software and hardware is also part of improving the signal-to-noise ratio (SNR). Some strategies are temporal gating to prevent ambient light, black box enclosures to block environmental photons, and chemical signal amplification methods like substrate recycling or enzyme stabilization. New research also uses CMOS sensor calibration and machine learning-based backdrop adjustment to obtain low-light signals more accurately. These new ideas all help make bioluminescent tests more dependable for use in clinical settings when resources are restricted [99].

### 3.4. Bioluminescence in Point-of-Care Testing

The integration of a bioluminescent system into diagnostic devices has paved the way for advanced POC testing methods. These applications range from detecting infectious diseases to monitoring biomarkers for chronic conditions with unparalleled sensitivity and specificity [100,101]. Compared to traditional diagnostic techniques that rely on costly equipment and time-consuming procedures, bioluminescence-based assays offer a promising alternative. The speed, simplicity, and accuracy of these tests make them ideal for rapid screening and early detection of various health conditions. There have been developments, specifically chimeric bioluminescent sensors that incorporate fluorescence and energy transfer mechanisms like BRET, which have enabled the high-resolution detection of biomolecular interactions [102]. One of the most critical benchmarks in evaluating POCT platforms is their ability to detect low concentrations of target analytes—referred to as the limit of detection (LOD)—and to do so with high specificity, minimizing false positives or cross-reactivity. Bioluminescence-based POCT, particularly those utilizing luciferase enzymes, offer a remarkable analytical performance due to their inherently low background signals, high signal-to-noise ratios, and rapid kinetic response [103,104]. The luciferase system can achieve LOD values in the femtomolar to low nanomolar range, outperforming many conventional technologies such as electrochemical (nanomolar to micromolar) and immunochromatographic assays (often in the micromolar range) [61]. The use of highly selective biorecognition elements, such as antibodies, aptamers, or even CRISPR-Cas complexes, enhances the specificity of these platforms, making them ideal for applications that demand accurate pathogen or biomarker identification [105]. Compared to electrochemical methods, which are valued for their portability and robustness but often suffer from signal interference and electrode fouling, luciferase-based assays provide cleaner and more reliable outputs [106]. Similarly, while immunochromatographic assays (e.g., lateral flow tests) offer ease of use and rapid results, they are typically qualitative and less sensitive, limiting their utility in early-stage or low-abundance detection [107]. Despite their promise, luciferase-based POCT systems face challenges such as enzyme instability, dependence on cofactors (e.g., ATP or coelenterazine), and the need for cold-chain storage. Recent advances in the use of metal–organic frameworks (MOFs), such as ZIF-8, have demonstrated significant potential in stabilizing and protecting luciferase enzymes from degradation due to temperature, pH changes, and proteolysis. These porous crystalline materials can encapsulate luciferase molecules, maintaining their bioactivity over extended periods and eliminating the need for refrigeration [66,108]. Together with developments in enzyme lyophilization and smartphone-based readers, these innovations position bioluminescent POCT as a powerful next-generation tool for rapid, sensitive, and decentralized clinical diagnostics, especially in low-resource or field settings [66].

Nonetheless, luciferase-based indicators of drugs (LUCIDs), bioluminescent sensor proteins that use luciferase to quantify analyte concentrations in patient samples, offer a novel mechanism for cost-effective POC biosensors [17]. Only a drop of the sample is spotted onto filter paper and using a simple point-and-shoot camera, the semisynthetic bioluminescent sensor can be engineered to selectively recognize a variety of drugs, including immunosuppressants, anti-epileptics, anti-cancer agents, and anti-arrhythmics. LUCID has shown an increased sensitivity in point-of-care testing through the non-invasive detection, sometimes of ultralow analytes, with rapidity. The latter feature has permitted the easy detection of low-level analytes and provided a close-to-real-time diagnostic result. Little or no equipment is required for assays based on LUCID; hence, this makes them highly relevant in resource-constrained settings. General portability and ease of transference between different diagnostic tests render LUCID versatile in applying to many diagnostics that assure accurate and quantitative data [109]. However, a significant factor in LUCID performing at its best is the platform that the POC device requires [102]. In the current field, there are a plethora of various platforms in which the sample is deposited and subsequently read by the detector in the assay. Some studies have used paper-based mediums, and others have curated a platform in which luminescence detection was improved.

**Figure 5:**
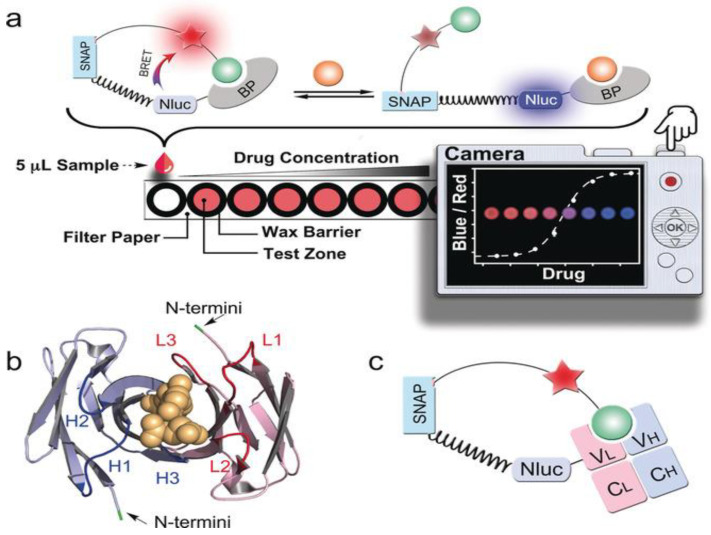
The design of LUCIDs for PoC diagnostics. (**a**) Schematic representation of the paper-based device. The LUCID is a fusion protein of SNAP-tag, NanoLuc luciferase (NLuc), and a binding protein (BP). SNAP-tag is labeled with a molecule containing a fluorophore (red star) and a ligand (green ball) that binds to BP. The filter paper was printed with wax circles, and the signal was collected by a digital camera. (**b**) The variable fragment of the methotrexate antibody (PDB ID: 4OCX) bound to methotrexate (yellow). The N-termini of both chains are indicated in green. The three CDRs (H1-3, blue) on the heavy chain (light blue) and three CDRs (L1-3, red) on the light chain (pink) are involved in antigen binding. (**c**) In Fab-based LUCIDs, the binding protein is an antibody Fab fragment. SNAP-tag and NanoLuc are attached to the light chain. The figure was reproduced from [109] with permission.

Paper-based medium POC devices have garnered attention for their advantageous features, including low cost, ease of use, quick turnaround times, and minimal consumption of reagents and samples [102]. Therefore, when trying to merge bioluminescence into POC devices, paper-based mediums seem to be a favored choice, especially in resource-limited settings. A paper-based method presented by Xue et al. genetically fused antibody fragments with NanoLuc luciferase and SNAP-tag, a self-labeling tag that allows researchers to attach fluorescent probes to proteins of interest. Once the target analyte binds to the antibody, the mechanism would disrupt BRET between the luciferase and fluorophore and quantify the analyte. These sensors enable POC drug level quantification in serum or blood by adding samples to paper and using a low-cost digital camera (Figure 5) [109].

**Figure 6:**
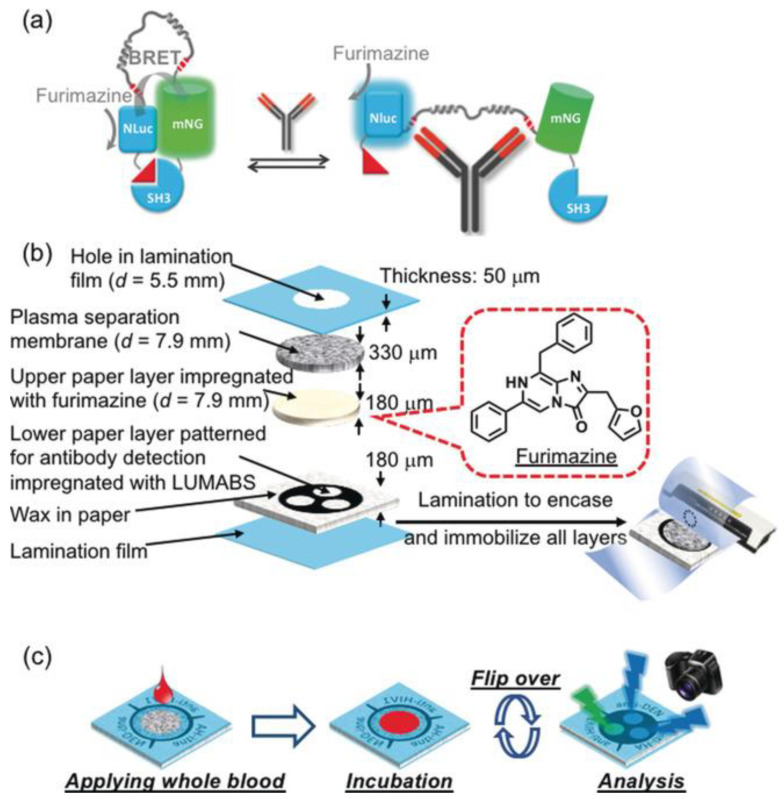
(**a**) Schematic of the LUMABS working principle with the “closed form” green light-emitting and the “open form” blue light-emitting protein sensor in the absence and presence of target antibody, respectively (NLuc = NanoLuc luciferase; mNG = mNeonGreen fluorescent protein). (**b**) Schematic of a multi-layer 3D-μPAD. All layers are kept together through lamination. (**c**) Schematic of the use of a 3D-μPAD for simultaneous detection of three different antibodies. The figure was reproduced from [110] with permission.

Similarly, another study used BRET-based antibody sensors in paper-based detection, allowing for sample volume-independent operation. Adding a single drop (20–30 µL) of the sample on a microfluidic paper-based analytical device (µPAD) and with a camera, it can detect three different antibodies (anti-HIV1, anti-HA, anti-DEN1) simultaneously in whole blood. This technology is a fully integrated 3D microfluidic paper-based analytical device (3D-µPAD) that incorporates newly developed bioluminescent BRET sensors, namely luminescent antibody sensors (LUMABS), for multiplexed antibody measurements in whole blood. In Figure 6, it can be seen that the device is built by stacking and laminating multiple layers of paper, creating a structured fluidic system that optimizes both detection and sample flow. The LUMABS sensor shifts its light emission from green to blue when an antibody binds, utilizing NanoLuc luciferase (NLuc) as the light source and mNeonGreen (mnG) as the energy receiver. Key components include a plasma separation membrane for blood filtration, an upper layer with detection reagents, and a lower antibody detection zone, all enclosed within a wax-sealed laminated structure for controlled fluid flow. Furimazine, as a substrate, moves through a layer containing immobilized LUMABS, generating a high-intensity signal without cofactors. The device simplifies fabrication by eliminating the need for precise paper alignment, allowing multiple target tests on a single platform. To measure antibodies, the device is flipped, allowing antibody to bind to the LUMABS, and the emitted light is captured using a digital camera. The closed lamination ensures a consistent sample volume, improving accuracy over traditional µPADs, which often produce variable signals based on sample size. Its compact design supports rapid testing with minimal whole blood, serum, or plasma providing reliable and reproducible results even with viscous samples. The design of the closed lamination constrains the volume of the sample absorbed in the device so that it may be detected consistently. This contributes to an improvement over traditional µPADs, which typically show different signal intensities relative to the applied sample volumes. A 3D-µPAD normalizes the absorbed sample volume and gives reliable and reproducible results. Discrete storage of luciferase-integrated BRET sensors and their substrate, luciferin, nearby provides for enhanced signaling efficiency, while patterning of the signaling layer allows multiplexed detection of targets. In that sense, the design of the device represents a considerable development toward point-of-care testing, offering speed, accuracy, and ease-of-operation assays. [110].

**Figure 7:**
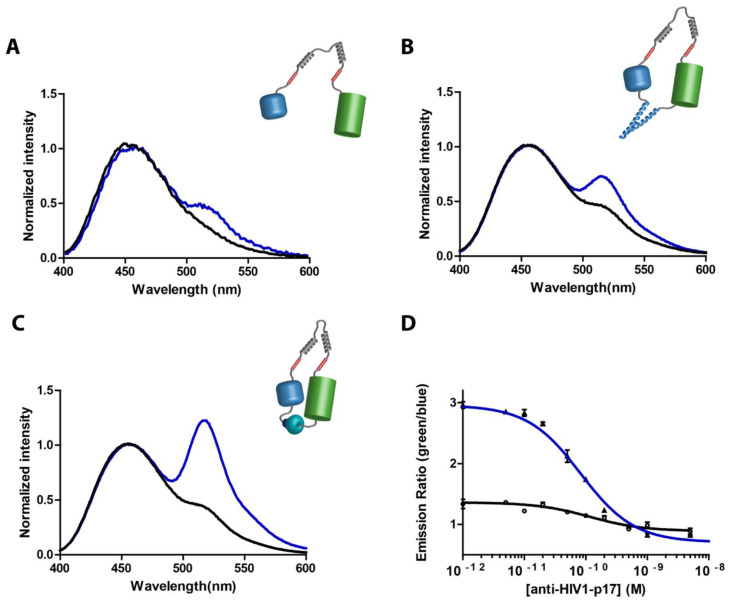
Characterization of various LUMABS designs. Luminescence spectra of LUMABS sensors in the absence (blue line) and presence (black line) of 1 nM anti HIV1-p17 antibody for (**A**) a sensor lacking helper domains, (**B**) a sensor with leucine zippers as helper domains (HIV-LUMABS-LZ1), and (**C**) a sensor employing the SH3-proline-rich peptide helper interaction (HIV-LUMABS-1). Spectra were obtained using 100 pM sensor protein. (**D**) Response of HIV-LUMABS-LZ1 sensor (Kd,app = 115 ± 41 pM) and HIV-LUMABS-1 (Kd,app = 83 ± 10 pM) to increasing antibody concentrations. These titrations were performed at a sensor concentration of 5 pM. All measurements were performed in a buffer composed of 50 mM phosphate, 100 mM NaCl, and 1 mg/mL bovine serum albumin at pH = 7.4. Data points are plotted as mean ± SEM (n = 2). The figure was reproduced from [111] with permission.

Another study combined the practicality of a smartphone-based POC device and the efficiency of BRET to create a new sensor platform. BRET measures the energy transfer between two luminescent molecules and requires a luminescent acceptor and donor. The luminescent donor is a molecule that undergoes bioluminescence, typically a luciferase enzyme, and the luminescent acceptor absorbs the energy from the donor and re-emits at a different wavelength. The single protein sensors present in LUMABS use NanoLuc in combination with the green-fluorescent acceptor protein mNeonGreen to generate an in situ signal against a dark background. This would reduce the need for external illumination or additional sample preparation. In this study, the researchers used antibodies against HIV-p17 (HIV1-p17, clone 32/1.24.89), hemagglutinin (HA-tag, clone 2-2.2.14), and dengue virus type-1 (dengue virus type 1, clone 15F3-1). In the absence of any antibody, these two domains within the sensor mechanism are kept tightly locked by auxiliary domains. The easy transfer of energy among the domains, due to the tight locking, is possible. A typical color light that was emitted was greenish-blue due to the efficient BRET between the domains (Figure 7). While the basic underlying principle of this process depends on BRET, the energy transferred between the two neighboring molecules leads to the emission of visible light. The presence of an antibody causes this molecule to bind to epitope sequences that are lying at the linker region between both domains. The point of interaction for the helper domain is in close proximity to the epitopes. The binding of the antibody breaks up the configuration and interaction of those helping domains so that the two detach from each other. So, the energy transfer is impaired or stopped altogether, and the color of the light emitted goes from green-blue to blue. This color change became a visible signal that indicated the antibody was present and attached.

Again, in this group, this well-chosen “switching” method was used, already introduced in several other successful sensor platforms for the real-time and very sensitive sensing of specific biomolecules. This strategy proved not only suitable for the qualitative confirmation of antibody binding but also as a quantitative test by calibration of the system. This system is very relevant for diagnostics, environmental monitoring, and biotechnological processes. It will allow one to detect the presence of a specific antibody or other analytes in complex samples and lead to a fast and direct optical readout of the signal, which can either be visualized directly in real time or quantified with a detector where needed. Using this domain-specific mechanism’s accuracy, investigators are now able to design sensors with superb sensitivity, giving the ability to trace even minute changes in molecular interactions, therefore enhancing the versatility of the system across a wide variety of applications. Furthermore, the LUMABS technology utilizes the ratio between two wavelengths for readout, making it less prone to variations in enzymatic activity, stability, and concentration. The use of a smartphone and the reduced susceptibility to variations in bioluminescent reading highlight the numerous potential applications LUMABS has in POC antibody detection [111].

On the other hand, another paper-based bioluminescent technology on POC was developed by Calabretta et al., based on bio-chemiluminescent detection with immunoassays on paper-based devices for point-of-care diagnostics. This allows for ensuring highly improved selectivity and sensitivity without loss in the simplicity of the light signal measurement that is intrinsic to bio-specific molecular recognition. Moreover, the device placed on paper can yield supplementary benefits of compactness, ease of use, and flexibility, which will add significantly to the attractiveness of portable accessible diagnostics [65].

**Figure 8:**
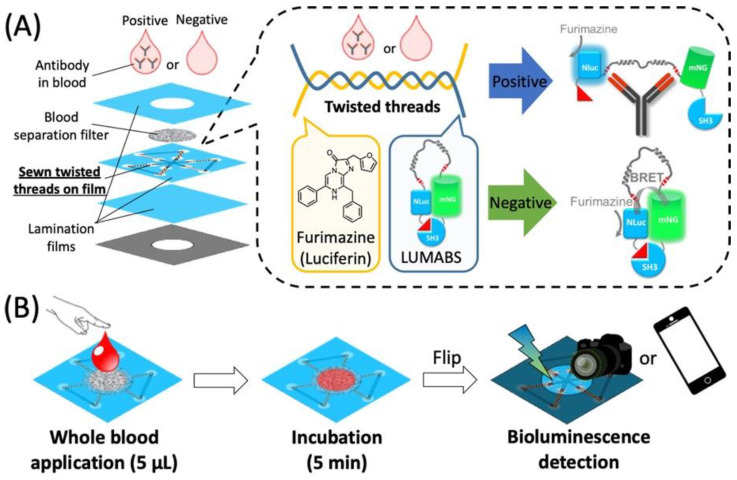
(**A**) Illustration of the bioluminescence reaction mechanism on μTADs, showing the separate dry deposition of LUMABS and its bioluminescent substrate (furimazine) on two intertwisted cotton threads; the emission of bioluminescence changes color from green to blue in response to a specific antibody. (**B**) Proposed μTADs analysis technique schematic: a total of 5 μL of whole blood sample applied to the device, bioluminescence signal captured by a digital camera or mobile phone camera 5 min after sample application. The figure was reproduced from [14] with permission.

Another study using a thread-based bioluminescent sensor for detecting multiple antibodies from whole blood (µTADs) has been developed using BRET. The device is made up of stacked layers which include a blood separation membrane and a plastic film with an embedded cotton thread. The BRET sensor proteins and substrate, furimazine, are redeposited onto the cotton thread (Figure 8). The BRET mechanism allows for a ratiometric readout of luminescent signals using a digital camera in a dark room or a smartphone camera with a 3D-printed lens adapter. The ability of μTADs to effectively detect three different antibodies was tested, anti-HIV, anti-HA, and anti-DEN, without any cross-reactivity or interference between the bioluminescence signals from one detection zone to the other. Whole blood samples spiked either with a single antibody or combination were applied onto the μTADs. Each device consisted of three individual sensors of LUMABS. These results reflect the unique bioluminescence signals given by each antibody, indicative of both the high selectivity of the BRET-based sensors and the efficiency of the hydrophobic barriers in signal-on/signal-off type assays, respectively. This allows simultaneous quantification with a sample volume of 5 µL of blood. The first demonstration of a user-friendly analytical instrument for one-step whole blood antibody assays was shown using bioluminescent sensor proteins attached to cotton thread substrate. General μTADs are very flexible for the detection of antibodies in minute quantities of whole blood via a BRET mechanism. They allow rapid detection with a mere 5 μL of blood, obtained through a simple prick on the finger, by immobilizing luciferase and luciferin in close proximity on intertwined cotton threads. The different parts of the thread can be coupled with diverse sensor proteins, allowing for 5 min signal six-plex detection without consideration regarding precise timing. In addition, the camera of a smartphone can be used to read out signals and μTADs are compatible with current data processing methods. This is an innovative technology for rapid, user-friendly point-of-care testing of one or more antibodies [14].

**Figure 9:**
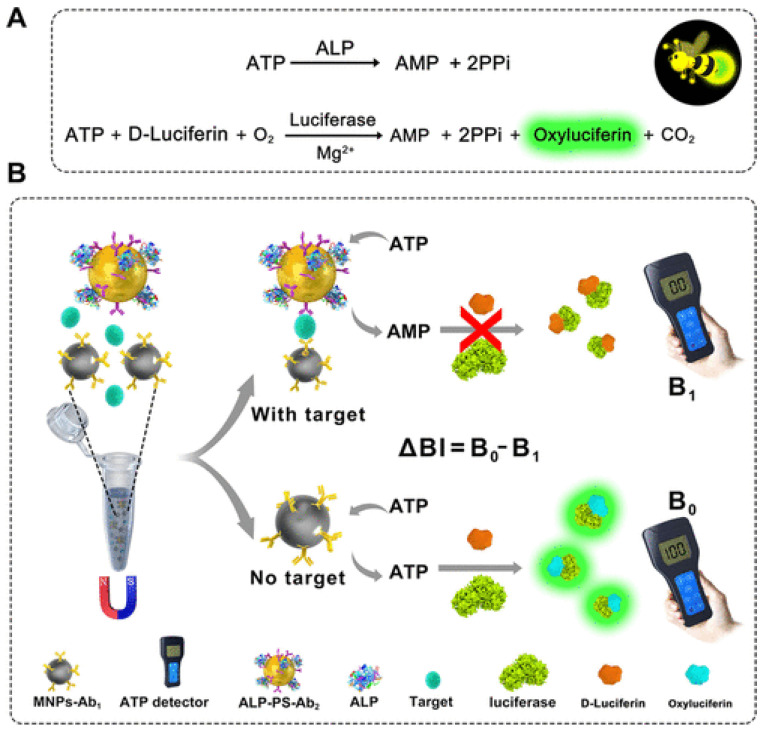
Scheme of the ABS for highly sensitive and quantitative detection of targets. (**A**) ALP can efficiently degrade ATP to AMP, which subsequently inhibits this bioluminescent reaction. In the presence of ATP, luciferase can catalyze the oxidation of luciferin into oxidized oxyluciferin and produce bioluminescence. (**B**) In ABS, the presence of target could be captured by the Ab1–MNPs and Ab2–PS–ALP to form the sandwiched PS–target–MNPs immuno-nanocomplex. After magnetic enrichment, the added ATP is dephosphorylated by ALP on the surface of PS, resulting in the change in the bioluminescence intensity (ΔBI) which is quantitatively measured by a portable ATP detector. The figure was reproduced from [112] with permission.

Many studies have branched out and have altered their detectors instead of developing novel mediums for increased detection sensitivity. While some POC devices use a point-and-shoot camera or a smartphone, others have diverged and started to rely on a portable detector. A study performed by Chen et al. revealed an innovative bioluminescent immunosensor (ABS) designed for POC testing that employs a dual-enzyme system of alkaline phosphate (ALP) and luciferase to obtain ultrasensitive and rapid detection of disease biomarkers in clinical samples. In the presence of a target biomarker, dephosphorylation of ATP by ALP-conjugated immuno-nanocomplex suppresses the bioluminescence in the ATP–luciferin–luciferase reaction (Figure 9). These ultra-sensitive ATP detections in the picomolar scale enabled the identification of this biomarker by monitoring the changes in bioluminescence intensity promoted by the ALP- and luciferase-mediated reactions. Quantitative results can be accomplished with a portable ATP detector at femtomolar-level sensitivity in less than 1 h, with minimal equipment. Thus, this ABS immunosensor has fulfilled three major demands: high sensitivity, speed, and portability. With rapid and accurate results, the ABS showcases potential for real-time, accurate biomarker analysis in clinical settings [112].

**Figure 10:**
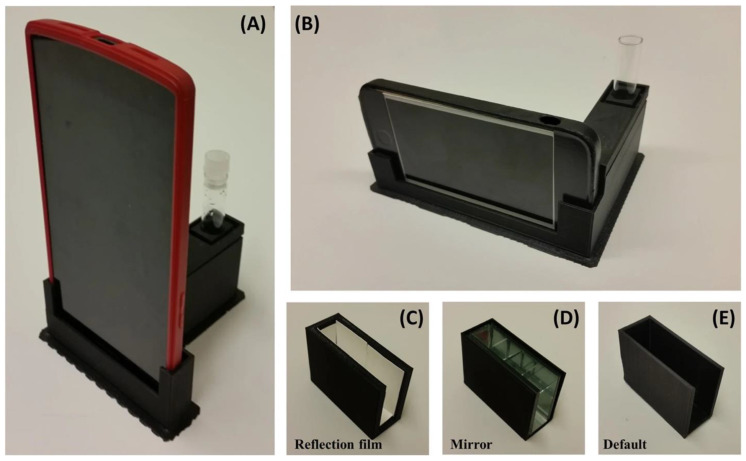
Detector chamber for BAQS: (**A**,**B**) shows BAQS for two different models; (**C**–**E**) displays a reflection film module, a mirror surface module, and default sample chamber, respectively. The figure was reproduced from [98] with permission.

Other methodologies of integrating bioluminescence in POCT include formulating a novel sensory-detection medium. One of the major challenges in using cell phones as detectors for bioluminescence-based systems is that they inherently lack the sensitivity of photomultiplier tubes, which have dedicated amplification circuitry. However, researchers are improving the sensitivity of smartphone-based detectors by enhancing the light collection efficiency and developing specialized software to reduce noise and improve the signal-to-noise ratio. For example, in a study conducted by Kim et al., a smartphone-based low-light detection for bioluminescence application called bioluminescent-based analyte quantification by smartphone (BAQS) was developed (Figure 10). The BAQS system is a bioluminescence detector that uses a smartphone with hardware and software created to enhance efficiency in the capture of photons. For this system, a newly designed sample chamber was employed along with sophisticated software-NREA that precisely measures the photons. The new sample chamber is covered with either mirrors or a reflective tape which increases the photon collection efficiency when compared to default ABS based sample chamber. Calibration is performed using an LED-based model system with variable light intensity controlled by neutral density filters. Then, the system was validated with live bioluminescent bacteria, i.e., Pseudomonas fluorescens M3A, which attained a sensitivity of approximately 7.9 × 10^6^ CFU/mL in both Android and iOS smartphones containing the special sample chamber. This embedding of software flexibility within strong hardware can potentially become a very attractive apparatus for technically versatile persons who can take advantage of several practical scientific activities right from a smartphone. This device can reduce background noise and enhance the signals from emitted photons [98]. Although the investigators did not demonstrate the device’s use for disease detection, the improvements they achieved in light collection efficiency will undoubtedly enhance its practicality in real-world applications.

**Figure 11:**
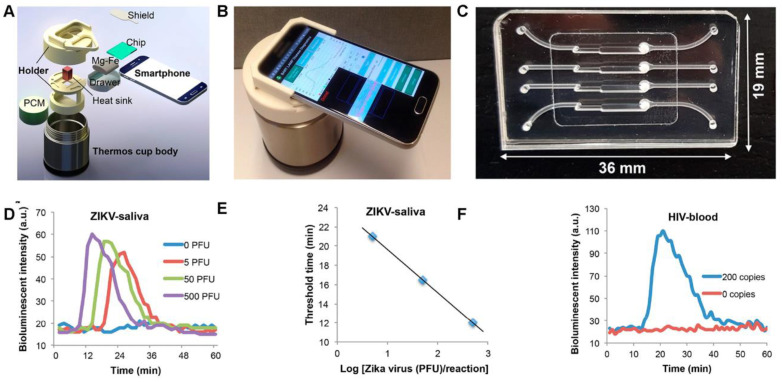
SCC platform for molecular diagnostics using BART-LAMP assay. (**A**) Detailed CAD rendering of the SCC. (**B**) A photograph of the assembled SCC. (**C**) A photograph of the detection microfluidics system. (**D**) Real-time monitoring of ZIKV amplification using BART-LAMP assay. (**E**) The quantitative analysis of the ZIKV virus. (**F**) HIV detection in blood. The figure was adapted from [114] with permission.

In 2010, Gandelman et al. introduced a real-time bioluminescent assay coupled with loop-mediated isothermal amplification (BART-LAMP), which continuously monitored the exponential accumulation of inorganic pyrophosphate (PPi) produced during nucleic acid amplification [113]. The BART system functions by converting PPi to ATP via ATP sulfurylase, enabling subsequent detection through thermostable firefly luciferase. This technology was applied to both the qualitative and quantitative detection of *Chlamydia trachomatis* in human urine, demonstrating 100% specificity and 95.6% sensitivity, with analytical performance comparable to that of quantitative PCR. Although the original study employed a custom-built instrument to detect bioluminescent signals, the authors also noted the potential feasibility of using a cellphone for signal acquisition. In 2018, Song et al. further advanced this approach by integrating the BART-LAMP system with a smartphone and a custom Android application to detect Zika virus (ZIKV) in human urine and saliva, as well as human immunodeficiency virus (HIV) in blood, all within 45 min [114]. The setup also included an insulated cup in which the heating for the isothermal application is generated by an exothermic chemical reaction (Figure 11). A phase-change material is used to regulate the incubation temperature whereas the smartphone app monitors the luciferin emission in real time quantifies the emission intensity and determines the target concentration. The whole system, dubbed “smart-connected cup (SCC)” provided a rapid, connected and quantitative molecular diagnostics platform.

Furthermore, recent works have altered the established mechanisms of POC devices, such as changing the sensory detection methods or the instrumentation to aid in increasing accessibility of POCT. Most POC devices rely on luminometers or fluorometers that require external excitation and possible further sample preparation, such as post-amplification steps [101]. All these additional steps may reduce the speed, sensitivity, and simplicity of the diagnostic test, while the aim is to achieve the opposite. Nevertheless, recent developments have shifted towards the fusion of complementary metal-oxide semiconductors (CMOSs) into POC devices, allowing compact, fast, and cost-effective solutions [115]. Most CMOSs are used for sensors that detect and process biological samples; therefore, POC devices that use microfluidic systems recruit the help of CMOSs as a detection module for signal processing. A recent study took the extra step and integrated real-time CMOS-based luminescence detection with impedance spectroscopy in a droplet microfluidics platform [115]. The luminescence chip eliminates the need for external excitation sources by detecting bioluminescence directly from the NanoLuc luciferase reaction occurring within the droplets in real time. It can sense optical signals from 38 nL droplets with a 6.7 nA/count resolution [115]. Additionally, by using a modular microfluidics system with integrated CMOS sensors, it offers a more compact, sensitive, and rapid approach to bioluminescence POCT, replacing oftentimes bulky apparatuses. This need for more simplistic, scalable devices that maintain sensitivity was echoed by Lomazzi et al., in 2020, where the traditional photomultiplier-tubes based system was questioned for a novel, more cost-effective option [116]. Photomultiplier tubes (PMTs) amplify very faint signals and are used for their reliability and accuracy in POC devices. However, Lomazzi et al. contended that PMTs tend to be bulky and are more expensive to other solid-state alternatives, such as silicon photomultipliers (SiPMs) [116]. After comparing detection limits, signal-to-noise ratio (SNR), and operating voltage, amongst other factors, SiPMs proved to be valuable in POC devices. While PMTs had a higher SNR in extremely low light intensities, SiPMs still maintained an acceptable SNR for biosensing applications, namely 100–1000 cps/mm^2^ [116]. Furthermore, SiPMs’ minimum detectable signal was in the order of 10^4^–10^5^ photons per second, which is also suitable for biosensing applications [116]. A considerable factor, however, is that PMTs consume hundreds to thousands of volts, whereas SiPMs operate at low voltages (30–60 V), making them more energy-efficient and easier to integrate into portable, battery-powered POC devices [116]. While PMTs are still the frontrunners for photon detection, SiPMs create a compelling case for themselves due to their portability, integration with POC devices, and economic advantage.

### 3.5. Advanced Bioluminescent Platforms

While POC devices are a relatively new concept, their integration into clinical, underserved settings is accelerating, driven by innovations that improve specificity, speed, and usability. Among the most promising advances are bioluminescent immunosensors, which harness the natural ability of immune-based tools to detect biomolecules rapidly and accurately.

One notable class of immunosensors is Quenchbodies (Q-bodies), which function by emitting fluorescence in response to antigen binding [117]. Q-bodies remain inactive, or “quenched,” until antigen binding, in which they undergo structural changes, releasing light [117]. However, in the past, Q-bodies were not prevalent in POCT due to their inability to function effectively in undiluted biological fluids and their storage and handling in paper-based POC devices were challenging [118]. Nonetheless, a recent study in November 2024 by Yang et al. created a BRET nano Q-body, a nanobody-based homogenous bioluminescent immunosensor, allowing one-pot detection for POCT [118]. The researchers fused NanoLuc luciferase and a cysteine-containing tag to the N-terminus of the nanobody, which was labeled with the fluorescent dye TAMRA. Then, the nanobody would recognize methotrexate (MTX), a chemotherapeutic agent, and produced a color change from the blue of the NanoLuc-catalyzed reaction to the red of TAMRA via BRET upon antigen binding. NanoLuc incorporation provides less light scattering and enhanced brightness in comparison to fluorescence provided by the Q-body [118]. Specifically, the paper reported a 7-fold increase in the emission ratio (TAMRA/NanoLuc) in a dose-dependent manner to MTX, proving its sensitivity [118]. Additionally, the researchers cemented the ability of BRET nano Q-bodies implementation with POCT, as the drug monitoring of MTX in biological fluids was successfully achieved after it was fabricated into a paper device, which remained stable for over a month at 25 C. Overall, the BRET nano Q-body is incredibly thermostable in organic solvents, reducing agents, and detergents, as well as biological fluids including milk, serum, and whole blood without dilution, with limits of detection of 0.50, 1.6, and 3.7 nM, respectively [118]. The stability and sensitivity of the BRET nano Q-body demonstrates its successful application in POCT and opens the door to a new realm of POC devices.

Parallel to immunosensor developments, CRISPR-based diagnostic tools have been developed, taking advantage of Cas effector proteins, specifically Cas9, Cas12, and Cas 13 [119]. CRISPR allows scientists to selectively modify DNA by cutting and inserting small pieces of desired DNA into precise areas with the help of the Cas proteins [119]. Following the COVID-19 pandemic, many have sought out CRISPR-based diagnostic tool as a prominent alternative to traditional nucleic acid-based detection techniques [120]. PCR-based methods, while immensely informative, provide serious setbacks such as requirements for expensive equipment, skilled personnel, and prolonged time, in contrast to CRISPR [120]. Therefore, combining CRISPR-based diagnostic tools with bioluminescent POC devices can provide an extraordinary number of benefits. A study completed in 2023 by van der Veer et al. developed a bioluminescent POC nucleic acid sensor (LUNAS) that detected pathogenic RNA/DNA using split luciferase complementation and CRISPR-Cas9 technology [121]. dCas9-based probes target specific sequences of double-stranded DNA (dsDNA), and when they bind to their target sequence, they combine the two inactive halves of NanoLuc luciferase, restoring its luciferase activity and indicating the presence of the target DNA. To enhance its specificity, as well as expand its use in field diagnostics, the LUNAS platform was combined with recombinase polymerase amplification (RPA), an isothermal amplification method [121]. Therefore, no extensive heating is necessary, and the target DNA is amplified by RPA at a constant temperature while maintaining attomolar sensitivity in a quick one-pot assay [121]. To ensure accuracy, a calibrator luciferase is included, providing the real-time monitoring of the RPA reaction due to its ratiometric readout. The authors then performed an RT-RPA-LUNAS assay to detect SARS-CoV-2 from samples with viral RNA loads of ~200 cp/uL and it was accomplished within ~20 min, highlighting its usefulness in POCT [121].

Together, these advances—BRET nano Q-bodies and CRISPR-Cas9 LUNAS systems—highlight the transformative potential of bioluminescence-based diagnostics in point-of-care testing. Whether through immune recognition or genetic targeting, these innovations significantly improve detection sensitivity, reduce equipment needs, and pave the way for rapid, user-friendly, and decentralized testing solutions. We have prepared a summary table detailing various bioluminescent POC technologies used for the detection of a wide range of analytes. The table outlines key information such as the analyte detected, sensitivity, and type of detector used. These advances demonstrate significant progress in making diagnosis faster, more affordable, and more accessible (Table 2).

The use of bioluminescence in the POC setting not only expedites the response to the management and treatment of disease but also showcases the technology’s versatility in detecting various infectious agents. One of the critical requirements for the application of bioluminescence assays at the point of care are their long-term storage with minimal loss of their functionality. This feature is important for the timely and reliable management of patients and the decisions related to their treatment. Long-term stability of such assays is difficult to achieve because bioluminescent reporters are proteins, and proteins per se are less stable than other biomolecules. Temperature fluctuations, proteolytic enzymes, and chemical instability may cause the degradation of proteins, including luciferases, which may result in the loss of bioluminescent activity. This limitation considerably diminishes their market availability, particularly in the case of bioluminescent (BL) immunosensors, for which only a limited number of instances are documented in the scholarly literature. For instance, a significant improvement was the development of a more stable bioluminescence assay by lyophilization containing all the necessary components for a luminescent reaction. This advantage broadens the prospects for reducing shelf-life problems of sensors, which work by the principle of bioluminescence, and their extensive entrance into the marketplace. Various researchers are trying to improve these continuously by inventing new materials and techniques that would make their operation easier for the end-user. This is important since only then can these lab-based devices be converted into practical and commercially viable products to meet the demands of modern POC applications. In this regard, it is expected that the increased availability of these bioanalytical instruments will lead to changes in analytical chemistry. More adoption of POC devices will make it cheaper and faster; it will make diagnosis routine; and it allows the fast and reliable testing right where it is needed. Such changes democratize diagnostics, ensuring wide access by the general population to the very latest instruments; once again, this will be a landmark regarding swiftness and efficiency in medical and environmental testing [122].

Many techniques are utilized to stabilize proteins. One common method is through lyophilization, in which a protein solution is frozen and then the ice is sublimated, leaving a stable, dry form of the protein. For example, lyophilized luciferase may be stored at room temperature and rehydrated with preserved activity [123]. Generally, the incorporation of stabilizers, for example, cryoprotectants such as glycerol or trehalose, buffer systems, and protein stabilizers like bovine serum albumin, can avoid degradation. Encapsulation techniques, such as biodegradable metal polymers or nanoparticles, shield the proteins from environmental stress. For example, encapsulation in poly lactic-co-glycolic acid (PLGA) nanoparticles may preclude the denaturation and degradation of luciferase [124]. Genetic engineering, such as mutagenesis for more stable variants of luciferase or fusion with a protein partner that would stabilize it, is possible for enhanced protein stability [125,126]. State-of-the-art stabilization techniques are needed in order to develop a trustworthy and practical bioluminescence POC assay.

In recent years, emerging nanomaterials have opened new avenues for protein stabilization, particularly for use in sensitive systems like bioluminescence-based point-of-care assays. Among these, metal–organic frameworks (MOFs) have garnered attention for their exceptional ability to protect and stabilize biomolecules. MOFs correspond to a class of porous materials constructed from metal ions coordinated with organic ligands within a crystalline structure that protects the encapsulated proteins against environmental perturbations. Based on this, through the incorporation of bioluminescent proteins into MOFs, a protective environment can be created to secure these proteins from temperature changes, proteolytic enzymes, and chemical instability. In particular, the encapsulation of luciferase within a zinc-based metal–organic framework enabled the preservation of its functional integrity during extended storage periods in order to realize point-of-care testing with uniform and reliable performance [66]. It is exactly these very properties—high surface area and tunable pore size—that make MOFs a material of choice for enhancing stability and efficacy in bioluminescent assays for use in clinical settings.

### 3.6. Metal-Organic Frameworks

**Figure 12:**
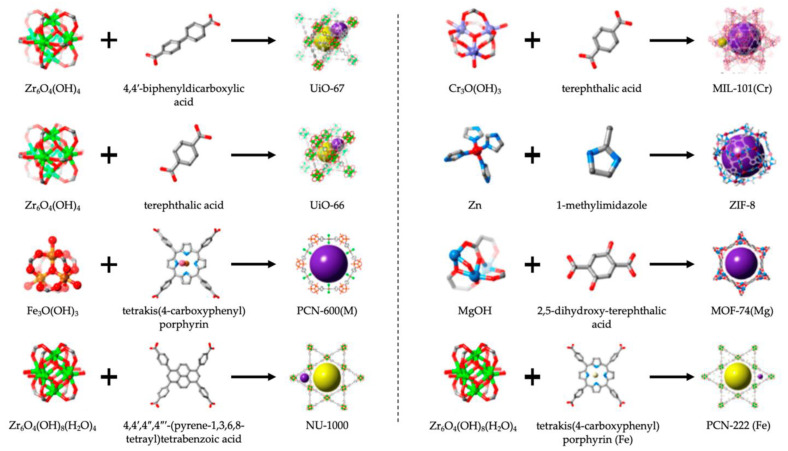
Various metal–organic framework architectures along with their respective metallic clusters and organic linkers. The figure was reproduced from [127] with permission.

Metal–organic frameworks (MOFs) are crystalline materials composed of metal ions or clusters coordinated with organic ligands (Figure 12) [127]. MOFs are porous materials with a high surface area, which makes them ideal for a wide range of applications such as gas storage, catalysis, drug delivery, and sensing [128,129]. To this day, the number of discovered MOFs exceeds 90,000, and further discoveries are ongoing [130,131]. MOFs have emerged as a transformative class of materials with vast potential across multiple scientific and technological domains. These porous structures are created through the interaction of metal ions with organic ligands, providing a high level of tunability that enables researchers to customize their properties for particular applications (Table 3) [132,133,134]. One important aspect involves the extended encapsulation and preservation of delicate substances like proteins [66,135,136]. MOFs demonstrate potential in effectively encapsulating unstable proteins for biological and cellular studies [137]. MOFs are frequently compared to a three-dimensional scaffold or framework in terms of their structure. Key features of MOFs include the following: (1) metal nodes, commonly metal ions of zinc, copper, chromium, and others; (2) organic ligands which are molecules with coordinating groups that link the metal nodes, forming the environmental factors that may cause denaturation, such as temperature fluctuations, pH variations, and the presence of proteolytic enzymes. Encapsulation within MOFs provides better stability to bioluminescent proteins with conserved bioactivity. MOFs can be prepared to bind through hydrogen bonds, van der Waal forces, or coordination bonds with the protein molecules to help retain its native structure. The encapsulation prolongs not only the shelf life but also the functional viability of such proteins; it also enhances their resistance to harsh conditions necessary for applications in industrial and medical fields. The application of MOFs to the stabilization of bioluminescent proteins opens new perspectives in the development of long-lasting and efficient bioluminescent systems for diagnostics, environmental monitoring, and therapeutic applications. Moreover, the intrinsic luminescence of some MOFs can synergistically interact with bioluminescent proteins to give enhanced or regulated light emission. This capability for framework and environmental tuning around the bioluminescent protein makes MOFs versatile in optimizing bioluminescence for target applications by affording increased precision over both emission intensity and duration.

**Figure 13:**
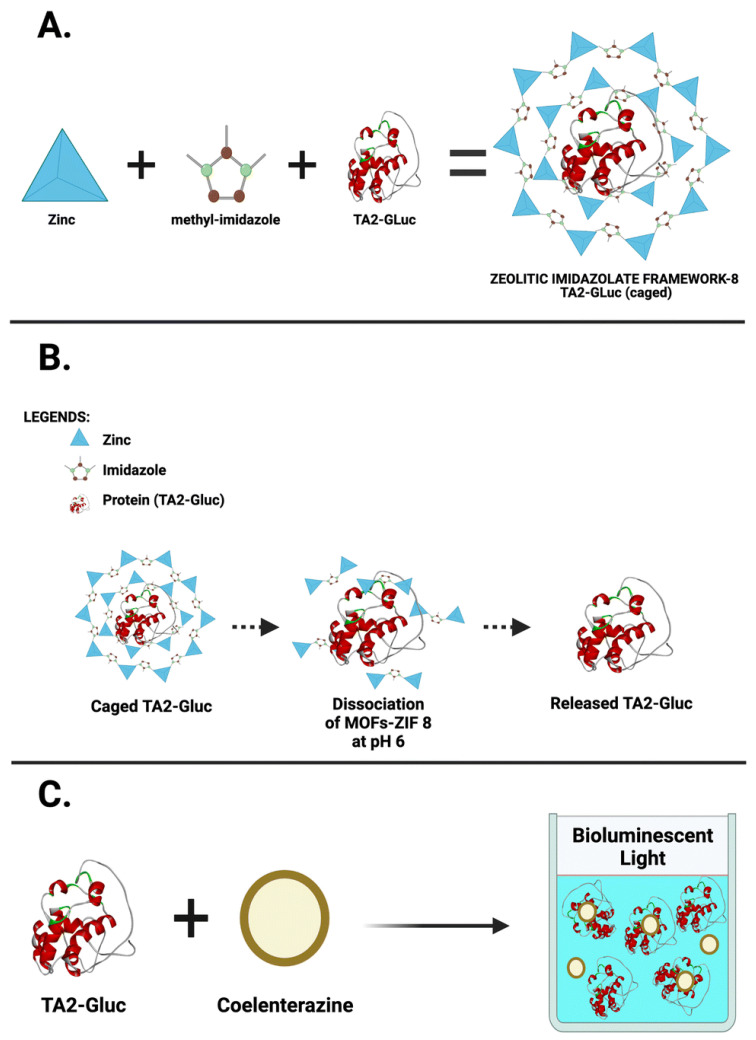
Metal–organic frameworks (ZIF-8) (**A**). Encapsulation of TA2-Gluc. (**B**). TA2-Gluc release at pH 6. (**C**). TA2-Gluc with its substrate coelenterazine producing bioluminescent signal. The figure was reproduced from [66] with permission.

The porous structure of MOFs allows the encapsulation of proteins, preventing their exposure to harsh external conditions that could lead to denaturation. Additionally, the interactions between the proteins and the MOF surface can stabilize the protein structure, preserving its bioluminescent properties [148]. The methodology enhances protection and the stability of enzymes, keeping their activity under conditions that usually lead to denaturation. Among the diverse MOFs, owing to the strong coordination of metal ions, is the ZIF-8 which exhibits superior chemical and thermal stability. Biomimetic mineralization was among the protective methods reviewed to avoid the inactivation of the activity of enzymes such as urease or horseradish peroxidase inside ZIF-8 under different conditions. In a study conducted in our laboratory, a bioluminescent immunoassay was developed. A laboratory-created universal reporter fusion protein, TA2-Gluc, was obtained by combining Gluc with the biotin-binding protein tamavidin2 (TA2) that in the presence of coelenterazine, created a bioluminescent light (Figure 13). This novel fusion protein is highly stable and can be stored at room temperature for over 6 months and used as a reporter in any assay that utilizes biotin–avidin binding. Thus, we utilized this fusion protein in a bioluminescent immunoassay to the detect severe acute respiratory syndrome coronavirus 2 (SARS-CoV2) antigen in various sample matrices [66].

In a study conducted by Lin et al., they designed a system that incorporated bioluminescence for the photodynamic therapy (PDT) of deeply seated cancer cells, wherein (d-Lu)PCN-224 utilized the bioluminescence resonance energy transfer (BRET) between PCN- 224 and d-Lu with a bioluminescence spectrum overlapping the absorption of PCN-224. This design epitomizes the huge potential for producing a large quantity of singlet oxygen required for the photodynamic destruction of cancer cells, without harming normal cells and avoiding the use of any external light. The results indicated that d-fluorescein was successfully loaded into the pores of PCN-224 at about 7.32 wt.%. It was observed that bioluminescence from d-fluorescein was effectively absorbed by PCN-224, which further confirmed that an internal energy transfer process took place. According to confocal imaging and cytotoxicity assessments, the system of (d-Lu)PCN-224 efficiently generated singlet oxygen, killing cancer cells in a targeted way. This MOF-based strategy may be applied more broadly for PDT in deep tissue cancers because it provides a potent, light-free therapy alternative [149]. Another study used MOFs to stabilize and immobilize bioluminescence in order to maintain an efficient and productive bioluminescent reaction. In this work, a well-recognized porous framework, MIL-53(Al), was used for the first time to support the immobilization of firefly luciferase. Stability was much improved with the immobilization process for all the stability values for the significant amount of enzyme loaded successfully over the MIL-53(Al) microporous matrix. The stabilization followed the adsorption method. MIL-53(Al) was characterized using different techniques of FT-IR, X-ray diffraction, FE-SEM, TEM, BET, and TGA in the study of several properties such as carboxylic groups, CO_2_ adsorption, crystallinity, purity, morphology, particle size and shape, specific surface area, and thermal stability. This further demonstrated that luciferase could bind to the surface of MIL-53(Al) without a linker, and the binding efficiency may increase with time. The method is quick, simple, inexpensive, and undertaken in mild conditions. These results would seem to indicate that MIL-53(Al) just might be a good choice for enzyme immobilization and can probably be extended to the others, such as proteins in biotechnological processes [150].

The integration of metal–organic frameworks into bioluminescence tests represents a significant advancement in the field of bioassay technology. Metal–organic frameworks (MOFs) possess a distinctive blend of exceptionally large surface areas, adjustable pore sizes, and remarkable stability. This characteristic enables the creation of synergistic combinations that boost the stability and effectiveness of bioluminescent proteins. MOFs, or metal–organic frameworks, not only safeguard biological samples from harmful environmental factors but also maintain the consistency and reliability of assay results over extended durations. Considering this fact, the innovation holds significant potential to enhance the reliability, precision, and practicality of bioluminescence assays undertaken in diverse clinical contexts. This will significantly enhance patients’ care and positively influence treatment outcomes.

## 4. Challenges and Future Directions

Point-of-care (POC) in vitro bioluminescence technologies, despite their considerable potential for rapid and remote diagnostics, face a range of challenges that require careful evaluation and innovative solutions. One of the main challenges lies in achieving the required sensitivity for detection at low analyte concentrations, given that bioluminescence signals can often be inherently weak [151]. This demands the development of highly sensitive detection systems and signal amplification techniques capable of discerning minute quantities of analytes in complex biological samples. An addition of charged-coupled devices (CCDs) improves the detection of low-intensity bioluminescence signals, especially back-illuminated CCDs which aid in photon detection efficiency [152]. In combination with optical enhancements, such as anti-reflective coatings and further reducing background noise, these modifications show great promise in providing the increased sensitivity desired.

Moreover, the tools and devices utilized in the in vitro bioluminescence tests for point-of-care (POC) applications need to achieve a delicate balance between compactness, portability, and user-friendliness, while also maintaining durability and precision. POC devices aim to assume efficiency and ease in their inner workings, allowing a wider demographic to use and benefit from them. The challenge is to create devices that are both technologically advanced and simply usable in various clinical environments without sacrificing analytical performance.

The intricate nature of biological samples at the point of care adds to the complexity of the situation. The samples may contain interfering compounds such as a reducing substance (ascorbic acid), oxidizing agent (chlorine), chemicals, drugs (acetaminophen), high levels of proteins, and many more that could affect the specificity of bioluminescence experiments. However, the use of bioluminescence based on its low background signal affords a better solution to the sample background/interference issue in terms of signal measurement.

The speed of analysis is a crucial aspect of POC diagnostics, requiring fast and dependable outcomes. Bioluminescence assays must be optimized to deliver prompt measurements while maintaining precision, in line with the usual urgency of POC situations. The detection of bioluminescence signal is very rapid; however, it requires the addition of a substrate. Therefore, integrating a feature of automated sample handling and addition of reagent in the POC devices remains of the uttermost importance to progress the field.

It is crucial to preserve the stability of bioluminescent reagents, such as luciferase, enzymes, substrate, or the bioluminescent cell itself, to ensure consistent test performance. In point-of-care (POC) settings, the need for stable formulations that can endure environmental variations becomes crucial. Current methods of stabilizing reagents include lyophilization, or free-drying, which stabilizes them at room temperature by removing water and slowing down degradation. Lyophilized reagents are typically available as lyobeads or lyocakes. However, due to space efficiency, meaning more doses of reagent can be lyophilized in a given area, effective heat transfer and a smaller bead size makes lyobeads a better choice for preserving reagents (10). This is important in POC settings, where efficiency is prioritized. Lyobeads speed up the reconstitution process by maximizing surface area-to-volume ratio. However, the inclusion of excipients, substances that aid in drug-delivery systems, in lyophilization can severely alter the reconstitution time and indirectly prolong the lyophilization process [153]. Excipients include stabilizers, bulking agents, buffers, and surfactants. Stabilizers remain of paramount importance as they can prevent changes during freezing or drying. Commonplace stabilizers include sucrose and trehalose due to their economic and biological advantages, yet novel polymer stabilizers such as polyethylene glycol (PEG), low-molecular weight polyvinylpyrrolidone (PVP), and cyclodextrins have shown promising results [153]. In comparison to sucrose, the novel polymer stabilizers consist of higher drying temperatures, allowing a shorter drying process coupled with intense drying conditions [153]. Regardless, the novel stabilizers cannot fully replace the use of sucrose or trehalose but combining them together can lead to a more time and cost-effective lyophilization. Additionally, it is important to seamlessly combine these factors to minimize sample manipulation, decrease the danger of contamination, and streamline the diagnostic process. Similarly, the cost issues are crucial in the general use of POC bioluminescence techniques. Aside from the expense of the reagents, the affordability of equipment and upkeep is vital in deciding the practicality and availability of certain diagnostic methods.

Standardization and quality control are crucial for assuring consistent and accurate findings in various point-of-care situations. Standardizing procedures and enforcing strict quality control measures is difficult because of the decentralized nature of POC testing environments. POC tests are performed by a variety of users, for different purposes, leading to potential inconsistencies.

Dealing with regulations is a major challenge. To obtain approval for POC bioluminescence assays, strict adherence to stringent standards for accuracy, reliability, and safety is necessary. Navigating these regulatory pathways requires cooperation among researchers, clinicians, and regulatory authorities to validate the effective assay performances and ensure compliance with medical regulations. This needs to be worked out as well with bioluminescent technology similar to any other POCTs.

Lastly, training users and interpreting information present additional challenges in point-of-care settings due to the diverse levels of expertise among individuals who may handle POCT. Creating bioluminescence tests that are accurate, user-friendly, and easily understandable by non-specialists is essential for effectively using these techniques in practical clinical situations. Adding visual cues to results or an explanation of potential results in the manual can provide casual users of POC devices an easier experience.

Addressing the challenges in point-of-care in vitro bioluminescence methods requires a multidisciplinary approach involving researchers, engineers, clinicians, and regulatory bodies collaborating to overcome these obstacles and realize the complete potential of bioluminescence for remote diagnostics.

## Figures and Tables

**Table 2:** Bioluminescence-based point-of-care technologies.

POC Technology	Analyte Detected	Sensitivity	Detector	Reference
LUCIDs (Luciferase-based indicator of drugs)	Immunosuppressants, Anti-epileptics, anti-cancer agents, anti-arrhythmics	Ultra-low analyte detection	Semisynthetic bioluminescent sensor with point-and-shoot camera	[17,109]
Paper-based NanoLuc-SNAP antibody	Drug levels in serum/blood	High	Snap-tag, BRET, low-cost digital camera	[109]
3D-µPAD (LUMABS)	Anti-HIV1, anti-HA, anti-DEN1 antibodies	--	BRET, digital camera	[110]
LUMABS with smartphone	HIV-p17, HA-tag, dengue virus type 1	High (qualitative + quantitative detection)	Smartphone camera	[111]
Bio-chemiluminescent paper device	General immunoassay targets, ATP	Improved selectivity and sensitivity	Bioluminescent luciferase + optical detector (smartphone camera)	[65]
µTADs (cotton thread)	Anti-HIV, anti-HA, anti-DEN1 antibodies	~5 µL blood, 5-min detection	Bioluminescent protein + smartphone with 3D lens adapter	[14]
ABS immunosensor (ALP + luciferase)	Disease biomarkers	Femto- to pico-molar sensitivity	Portable ATP detector	[112]
BAQS (Smartphone-based bioluminescent analyte quantification)	*Pseudomonas fluorescence* (live bacteria)	~7.9 × 10^6^ CFU/mL	Smartphone + custom software and sample chamber	[98]
BART-LAMP (Bioluminescent Assay)	*Chlamydia trachomatis* in human urine	95.6%	Thermostable firefly luciferase	[113]
BART-LAMP with Smartphone Integration	Zika virus (ZIKV) in human urine and saliva, HIV in blood	Not specified for ZIKV or HIV	Smartphone app (monitors luciferin emission), ATP sulfurylase converting PPi to ATP	[105]
CMOS + impedance spectroscopy	NanoLuc bioluminescence in droplets	6.7 nA/count resolution (very high)	Integrated CMOS sensor in microfluidic chip	[115]
SiPM-based system	General photon detection (for biosensing	10^4^–10^5^ photons/sec	Silicon photomultiplier (CiPM)	[116]
BRET nano Q-body	Methotrexate (MTX)	0.5, 1.6, 3.7 nM (milk, serum, whole blood))	Paper + bioluminescence (NanoLuc → TAMRA)	[118]
LUNAS (CRISPR + Luciferase)	Pathogenic RNA/DNA (e.g., SARS-CoV-2	Attomolar sensitivity	Ratiometric luciferase, portable detector	[121]

**Table 3:** Classification of MOFs.

Class	Examples	Definition	Use	Reference
Iso-reticular MOFs (IRMOFs)	IRMOF-3, IRMOF-n (n = 1–16)	Octahedral microporous crystal material assembled by [Zn_4_O]^6+^ and different ligands	IRMOF-3, recognition of 2,4,6-trinitrophenol in wastewater	[133,134,138]
Zeolitic Imidazolate Frameworks (ZIFs)	ZIF-8, ZIF-90, ZIF-L, ZIF-71, ZIF-67, ZIF-7	Zeolite structure formed by combining transition metal ions and imidazolyl ligands	ZIF-8, detection of HIV-1 DNA	[133,134,139]
Porous Coordination Networks (PCNs)	PCN-333, PCN 224, PCN-222, PCN-57	Stereo octahedrons having a three-dimension structure that has a hole–cage–hole topology	PCN-222, electrochemical sensor to detect DNA	[133,134,140]
Materials Institute Lavoisier (MILs)	MIL-101, MIL-100, MIL-53, MIL-88, MIL-125	Synthesize using various elements that have valence electrons and an organic compound containing dicarboxylic acid ligands	MIL-101 (Cr), Resistive humidity sensors MIL, chemical sensors to immobilize, proteins, quantum dots, and other constituents	[133,134,141,142,143]
Porous Coordination Polymers (PCPs)	Prussian blue—PCP, PCP Zn(NO_2_-ip)(byp) ***	Synthesize by self-assembly of carboxylic acid, pyridine, and their derivatives as ligand (PBU) * and transition metal ions (SBU) **	PCP Zn(NO_2_-ip)(byp), organic vapor sensor	[133,134,144,145]
University of Oslo (UiO)	UiO-66(Zr)	Based on carboxylic acid (PBU) and Zr_6_(µ_3_-O)_4_(µ_3_-OH) (SBU)	Supercapacitor electrode material	[134,146]
Other MOFs	Northwestern University (NU), Pohang University of Science and Technology (POST-n), Dresden University of Technology (DUT-n family), University of Nottingham (NOTT-n), Hongkong University of Science and Technology (HKUST-n), Christian-Albrechts- University (CAU-n family), and Biological metal–organic frameworks (Bio-MOFs)	Recently discovered MOFs	HKUST-1, used as chemical sensors for detecting dopamine,	[133,134,147]

* Primary binding unit; ** secondary binding unit; *** 4,4′-bipyridine.
